# Motor Program Transformation of Throwing Dart from the Third-Person Perspective

**DOI:** 10.3390/brainsci10010055

**Published:** 2020-01-18

**Authors:** Alexey Tumialis, Alexey Smirnov, Kirill Fadeev, Tatiana Alikovskaia, Pavel Khoroshikh, Alexander Sergievich, Kirill Golokhvast

**Affiliations:** 1 NTI Center for Neurotechnology and VR/AR Technologies, Far Eastern Federal University, Vladivostok 690922, Russia; smirnov.aserg@dvfu.ru (A.S.); fadeevk.fefu@gmail.com (K.F.); 2 Far Eastern Scientific Center of Russian Academy of Education, Far Eastern Federal University, Vladivostok 690922, Russia; alikovskaia.ta@dvfu.ru (T.A.); khoroshikh.pp@dvfu.ru (P.K.); sergievich.aa@dvfu.ru (A.S.)

**Keywords:** body schema, darts, kinematic, motor program, third-person perspective

## Abstract

The perspective of perceiving one’s action affects its speed and accuracy. In the present study, we investigated the change in accuracy and kinematics when subjects throw darts from the first-person perspective and the third-person perspective with varying angles of view. To model the third-person perspective, subjects were looking at themselves as well as the scene through the virtual reality head-mounted display (VR HMD). The scene was supplied by a video feed from the camera located to the up and 0, 20 and 40 degrees to the right behind the subjects. The 28 subjects wore a motion capture suit to register their right hand displacement, velocity and acceleration, as well as torso rotation during the dart throws. The results indicated that mean accuracy shifted in opposite direction with the changes of camera location in vertical axis and in congruent direction in horizontal axis. Kinematic data revealed a smaller angle of torso rotation to the left in all third-person perspective conditions before and during the throw. The amplitude, speed and acceleration in third-person condition were lower compared to the first-person view condition, before the peak velocity of the hand in the direction toward the target and after the peak velocity in lowering the hand. Moreover, the hand movement angle was smaller in the third-person perspective conditions with 20 and 40 angle of view, compared with the first-person perspective condition just preceding the time of peak velocity, and the difference between conditions predicted the changes in mean accuracy of the throws. Thus, the results of this study revealed that subject’s localization contributed to the transformation of the motor program.

## 1. Introduction

Throughout life, subjects develop a perception of their actions from the first-person point of view. At the same time, in some situations, the subject is given the task of regulating his own actions when he perceives himself from an outside perspective, for example, in a mirror during rehearsals and trainings, while controlling an avatar in computer games using virtual reality (VR) and motion capture, or controlling a remote robot that repeats the operator’s actions. 

In the first-person perspective the person looks from the position of localizing himself within the borders of his own body. In the third-person perspective (3PP) subject’s perception occurs from the position of localizing oneself in space outside the boundaries of one’s own body, for example, when subjects see himself reflected in a mirror.

The first-person perspective (1PP) facilitates the transfer of information between visual and motor systems due to direct coincidence in measurement between the body image and the corresponding representation of an action. The result of this coincidence is expressed in faster and more accurate subject’s reactions [1]. Changing the perspective has negative effects on memory [2,3] and the intensity of emotional reactions decrease [4], the initiation and execution of an action slow down, its accuracy also decreases [5,6]. These changes are associated with a change in the localization of a subject and, as a result, cause several mismatches in the regulation of the execution of an action. There is a conflict between the motor program and visual reafferentation [7]. Some researchers have proposed the presence of two levels of regulation when the perspective changes [8,9,10]. Level 1 is associated with the automatic calculation of the perspective—at what angle the other person looks and what area of the visual scene he sees. The priority of perception from the first-person perspective at this level was not found [10] suggesting that the lability of perception of perspective is necessary for social orientation. For example, the performance of an action by another person causes a spontaneous change in the perspective [11], that is, perspective taking is influenced by the attribution of intentions to others [12]. Level 2 is associated with the explicit stimuli discrimination from a third perspective [8] and the priority of the first-person perspective is manifested at this level.

The self-perception from the third-person perspective is also referred to as an out-of-body experience or disembodiment [13]. This phenomenon has been investigated through the sensory conflict [14], but attributing authorship of movements also affects the ability of identification [15]. For example, a study [16] found that a conflict between the performance of a movement and its visual perception results in the feeling of authorship (or agency) of the action and ownership (or embodiment) of the body decreases.

Another conflict in the perception of one’s own actions from the 3PP arises between the proprioceptive and visual perception of action [17]. Numerous studies have been conducted using lenses that cause a mismatch between the actual hand position and its visual perception [18,19]. However, in these experimental models, the subject remains in the same localization inside the borders of his own body and therefore, following a small number of attempts, the impaired coordination of movement successfully restores. In the 3PP, the self-localization as a source of activity is changed. A few studies analyzed the accuracy and temporal parameters of movement when the subject performed the actions from the 3PP [5,6] and, as far as we know, an analysis of the kinematics was not previously conducted.

Perception of body parts in space is defined as body schema. Electroencephalographic source localization studies show that changes in body schema through the third-person perspective is associated with the activation of right temporo-parietal junction [20] and theta-burst magnetic stimulation of this region results in facilitation of the perspective changes [21]. Easier changes of the perspective, from first to third-person view, are also obtained in subjects with higher heartbeat perception [22], in subjects of Western culture and in women [23].

Theoretical frameworks that explain the change in motor control associated with altered body schema in third person point of view are as follows.

Reinforcement learning account suggests that action control is based on the prediction of long-run future consequences, such that motor control is aimed at maximizing rewards and minimizing negative outcomes. The dopamine neuromodulator provides basal ganglia target structures with phasic signals that convey a reward prediction error, or difference between the real consequences of action and the planned results, that can influence learning and action selection [24].

In motor control theory [19,25,26], the inner motor model includes two models, an inverse model and a forward model. Inverse models transform desired goals into a plan to accomplish them. To learn an inverse model, one needs to evaluate the consequences of action, which may be sensed with delays and noise. So, the brain has developed the predictions, which can be achieved by using forward models that simulate the outcomes of a given plan. Forward models are used to give instantaneous predicted feedback that provides an estimate of the state of the controlled effector, and thus to overcome the significant delays of real sensory feedback. When a real sensory feedback does arrive, it can be combined with such predicted feedback to compensate for limitations and the noise of the sensory systems. Thus, forward models are used to generate sensory error signals (predicted feedback minus real feedback), which can guide learning of inverse models. The other proposed role for the forward models involves anticipating and cancelling the sensory effects of self-initiated actions from the incoming sensory stimuli.

According to the predictive coding account [27], brain develops a prediction model about sensory inputs, trying to maximize model evidence and to suppress the deviation from this model, defined as a prediction error or a surprise. An action depends vicariously on sensory states so that sensory prediction errors generate motor command signals and predicts an intimate anatomical relationship between primary motor and sensory systems. On the other hand, an action suppresses sensory prediction by changing the motion of sensory states so that they conform to conditional expectations. This formulation suggests that motor control may not be the selection of an action but an emergent property of the predictive coding.

Studies of perspectives of perception often use static images of other people [11] or parts of their body [1,6,12,17], as well as avatars [4,8,9,10,28], and do not pay attention to studying the regulation of actions when the perspective of perception is being changed. On the other hand, studies of the perception of one’s own actions [18,29,30,31,32] do not include the perception from a third-person view. Jeannerod [15] has shown that the perception of one’s own actions (that is, the correspondence between the motor program and the visual reaffirmation) is an independent self-awareness factor, but this study has not analyzed the kinematics of movements, but only a subjective assessment of an identity. In the current study, we investigated the kinematics of the movement of throwing darts in various conditions of visual perception. The goal of the game of Darts is to hit a spot in the central region of the target (bull’s eye). A typical sequence of events consists of aiming, swing, acceleration, release of the dart and completion of the throw/getting feedback about the result. During the performing of dart throws, we recorded the position and speed of the hand by using motion capture sensors. In the experiment the subjects performed dart throws in the first and third-person point of view conditions. To simulate the perception from the 3PP, a video feed from the camera, installed behind and above the subject, was directed into a head-mounted display (HMD), as a result, the subjects directly perceived themselves and their actions from the outside perspective. Thus, unlike previous studies that used a mental rotation to model the perception from the third-person view, and analyzed the response time as well as accuracy to assess the difference between perspectives [1,6,8,9,10,12,17,28], in this study we used a motion capture of subject’s movement while he perceived himself from the 3PP. Such a research paradigm allows separating the subject’s body and the localization from which visual perception is carried out and to study the regulation of movement in this condition. In healthy subjects, the border between the unrecognized changes in the angle, induced automatic motor correction, aware of changes of the angle, and evoked consciously controlled movement corrections, lies around 14–15 degrees [33,34]. In the current study, the camera angle was equal and greater than 20 degrees, which should have caused the subjects to activate the mechanisms of the consciously controlled correction of the hand trajectory when performing dart throws, accompanied by a decrease in their identity with their own body. 

We expected that the third-person perspective would evoke a decrease in the accuracy of actions, compared with the 1PP condition, in accordance with the data of Watanabe and Higuchi [6]. In accordance with the data from Gorisse et al. [19], we expected the decrease in the amplitude and speed parameters of the movement performance in the third-person perspective condition compared with the 1PP condition. 

## 2. Materials and Methods

### 2.1. Subjects

The subjects were 31 students and staff of the Far Eastern Federal University. The data of three subjects were removed due to technical errors during data registration. The final number of subjects was 28 (age = 22.2 ± 3.13; body mass index = 22.06 ± 3.21; number of males, *N* = 14; years of education = 14.4 ± 2.22, height = 172.1 ± 8.70). Subjects had no or little (few times in their lives) experiences in playing darts and no sports activities addressed accuracy in motion (shooting, throwing, cybersport or others). All participants were volunteers. Subjects have no neurological or mental abnormalities according to the self-report. They did not consume alcohol 24 h before the study and tonic beverages three hours before the study. All subjects were right-handed because in the 3PP condition we changed the camera’s location to the right. The study was conducted in accordance with the Helsinki Declaration and was approved by the Ethics Committee of the Far Eastern Federal University. All subjects signed an informed consent.

### 2.2. Procedure

Upon arrival at the laboratory, subjects completed an informed consent. Then they performed 10 training dart throws. After that, they put on a Perception Neuron 32 suit to capture the movement. The suit version contains the following sensors: lower back, chest, left and right shoulder, right arm, right forearm, right hand and each finger of the right hand. This set of sensors made it possible to receive data from all parts of the limb participating in the throwing. However, finger movement data had to be excluded from the analysis due to insufficient equipment accuracy. The sampling rate was 120 Hz. The motion capture suit was connected to the computer via a Wi-Fi network. The computer running the application had an Intel Core I9-9700X @ 3.30GHz CPU, 64 Gb RAM and a Nvidia GeForce RTX 2080 Ti GPU.

After the calibration of the motion capture suit, the subjects were instructed on the rules for throwing and asked to put their feet on the pre-marked lines on the floor. The purpose of these lines was to keep all the subjects in the same position throughout the experiment.

**Figure 1:**
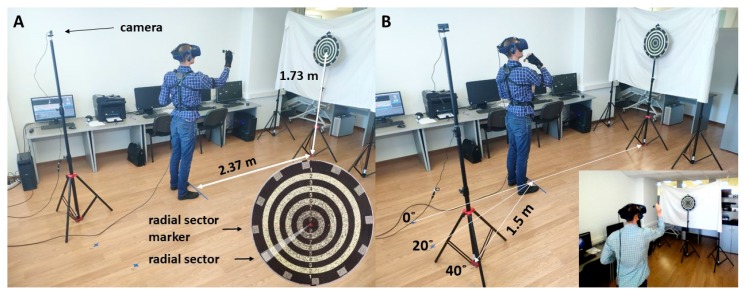
Method of the study. Subjects wore the motion capture suit and threw the darts from the first and the 3PPs. (**A**) dartboard location relative to the subject, the insert shows the dartboard with the radial sector and radial sector marker. (**B**) The relative to the subject location of the camera used to simulate perception from a 3PP. The insert shows the image taken from a virtual reality (VR) headset when the subject threw the darts in a third-person perspective with a camera placed at a 40° angle position.

The dartboard, 450 mm in diameter, consisted of concentric black and white rings with a score from 1 on the periphery to 10 in the center, as well as radial sections clockwise from 1 to 36 of 10°. The dartboard was placed at a 2.37 m distance and its center was at a height of 1.73 m in accordance with the rules of the World Darts Federation ([35]; Figure 1A). The plastic darts had a metal tip, the weight of the dart was 16.5 g.

Subjects performed throws with their leading right hand. The accuracy of the hits was recorded in accordance with the dartboard rings in the range from 1 to 10, as well as a radial position from 1 to 36. The missed throws were scored as zeroes.

Participants were asked to throw darts at the target in four conditions—from the first-person perspective and from the 3PPs with the camera moved on 0°, 20° and 40° to the right relative to the direction of the throw. The 1PP condition was performed first. Then, three conditions with a 3PP were given for each participant in random order, without a gradual increase or decrease in the angle of perspective. In each of the four conditions, subjects performed 40 throws of five darts per series. After each throw, the first experimenter announced the accuracy of the throw and the radial sector and the second experimenter registered it. After every five throws, the first experimenter cleared the dartboard of darts. In total, every subject had to perform 160 throws. 

To simulate the 1PP, the built-in HTC Vive Pro HMD frontal cameras were used. To simulate the 3PP, we used a projection of a video feed from an external high definition wide-angle USB-camera Genius WideCam F100 into the VR HMD. The camera was located behind the subject at a distance of 1.5 m and about 20 cm higher than the subject’s height, producing the vertical viewing angle deviation of about 5 degrees. The location and direction of the camera were chosen so that the subject could see the target, his own body down to the waist and the trajectory of the arm during the throws (Figure 1B). In the current experiment, the 3PP was presented in three different by the angle of the camera conditions. The camera location strictly behind the subjects (zero degrees’ condition, 3PP0) created the condition of the 3PP without the distortion of the horizontal perspective angle. Moving the camera at 20 and 40 degrees (3PP20, 3PP40) to the right created the increased 3PP angle of perception.

### 2.3. Accuracy Analysis

Analysis of the accuracy of hits was carried out for each condition (1PP, 3PP0, 3PP20 and 3PP40) and only for throws that hit the target.

The hits were registered in the polar coordinate system and then were transformed to the Cartesian coordinate system where 1 radial section was equal to 10° and 1 dartboard ring had a thickness of 22 mm (except for the 10-point outer ring, which had 44 mm thickness). The calculation was carried out in accordance with the formulas: x = r × cosφ, y = r × sinφ, where x and y are the Cartesian coordinates, r—target ring, φ—radial section. 

For each subject in each condition, the average absolute accuracy and relative accuracy in millimeters along the OX and OY axes in the Cartesian coordinate system and the number of hits were calculated. Absolute accuracy was calculated as the mean distance from the center of the dartboard. Relative accuracy was calculated as the standard error of the mean (SEM) accuracy.

### 2.4. Kinematics Analysis

**Figure 2:**
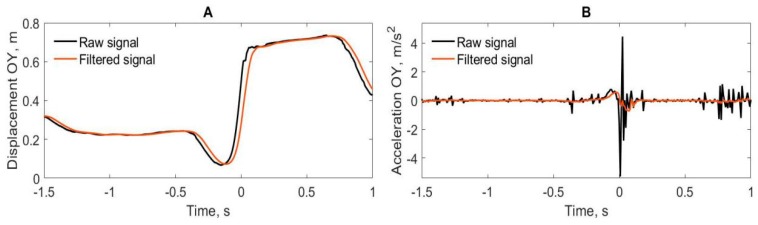
The example of the right-hand movement along the OY axis in Subject No. 1 in the first-person perspective (1PP) condition. (**A**) Raw and filtered data of the hand displacement. (**B**) Acceleration data obtained from the raw signal and acceleration obtained from the filtered signal. Time 0 represents the moment of peak velocity of the right hand moving along the OY axis (along the direction of the throw).

For the analysis of kinematics, the right-hand sensor was selected, as well as a sensor from the sternum of the spine. All motion recordings contained some level of noise, which is a high-frequency change in the signal that is not caused by the movement of the subject. The presence of the high-frequency noise made the acceleration analysis difficult, so, we filtered the signal. Figure 2A shows the raw displacement signals with the presence of high-frequency noise and smoothed filtered signal. Figure 2B shows the hand acceleration in the same period on movement.

The displacement of the right hand along the OX, OY and OZ axes and the sensor quaternion of the torso around the OZ axis, described the rotation of the body, were exported to MATLAB 2017b. Then, we used a moving-average filter with a window width of 10 points to remove the high-frequency noise from the data. The velocity of movement was calculated as the first time derivative for each axis. The acceleration of movement was calculated as the second time derivative for each axis. For each condition, the maximum velocity of the hand was identified along the OY axis, that is, perpendicular to the axis of the body and in the direction of the throw. This parameter was chosen because it had a clearly defined peak, which reflects the event of releasing a dart [36]. Other researchers describe the difference in peak velocity and dart release at 2–11 ms [37], which corresponds to about 1 frame of a mocap suit recording. Peaks were identified using the built-in findpeaks function. Then the movements were analyzed in according with the perspective conditions: from the 1PP and of 0°, 20° and 40° from the 3PP. Next, the data were cut into segments from −1.5 to +1 s relative to the moment of peak velocity of the hand. 

To analyze the kinematics, we used data on the displacement, velocity and acceleration of the right hand along the OX, OY and OZ axes. The data of the body rotation around its OZ axis throughout the dart-throwing was analyzed using quaternion. Additionally, we calculated the angle of the hand movement in the period of throwing. For this, we took 12 points (or 0.1 s of the registration time) in the OX and OY axes of hand movement preceding the time point of peak velocity of the hand along the OY axis. In that period, participant’s hand moved more or less linearly from backward move point to the dart release point. Later points, followed dart release, were not affect the dart flying angle and were not used in the analysis. The angle was calculated in every two neighboring time points according to formula Angle =$\;arctan\left|\frac{x2-x1}{y2-y1}\right|$, where *x*_2_, *y*_2_—the position of the hand along the axes OX and OY at one moment in time, *x*_1_, *y*_1_—the position of the hand along the axes OX and OY at the previous moment in time.

All throwing movements that did not result in a goal hit were excluded from the analysis. The trajectories were averaged for each subject in each condition: 1PP (1PP), third-person perspectives in 0° condition (3PP0), 20° condition (3PP20) and 40° condition (3PP40).

### 2.5. Statistical Analysis

For the statistical analysis of the differences in the number of hits between conditions a repeated measurements ANOVA with the factor condition (1PP, 3PP0, 3PP20 and 3PP40) was used. Student’s *t*-test for paired-samples was used to analyze paired differences in number of hits between conditions.

Analysis of hits accuracy was conducted in three variants. Firstly, one-sample Student’s *t*-test was used for analyzing the deviance of mean hit accuracy from zero in axes OX and OY in each condition. Secondly, repeated measurements ANOVA with throw conditions (1PP, 3PP0, 3PP20 and 3PP40) and axes (OX and OY) was used to analyze the effects of condition on absolute accuracy. Student’s *t*-test for paired-sample *t*-test was used to analyze paired differences. Third, two-sample F-test was used to check the difference in relative accuracy between each pair of conditions. 

Effect sizes of significant results are reported as proportion of explained variance (partial eta squared, $\eta_{p}^{2}$). In case the assumption of sphericity was violated, the degrees of freedom for all ANOVAs were corrected by Greenhouse-Geisser epsilon (*ε*) and Bonferroni corrections were applied to correct the α-levels.

Statistical analysis of kinematic data was carried out in the SPM1d package [38,39]. This package is designed for the analysis of kinematics on the basis of scripts for the analysis of three-dimensional tomography results in the SPM package. It uses random field theory (RFT), which is charged with solving the problem of multiple comparisons. Random field theory is superior to other correction methods since it conducts inference based on the height and the size of connected clusters that remain following the suitably high SPM{t} thresholding (e.g., *t* > 3.0). Precise probability computations additionally depend on field smoothness and search space morphology. A key point is that a large suprathreshold cluster is the topological equivalent of a large univariate *t*-value.

So, SPM1d allows analyzing trajectories for the entire duration of the movement. It also provides an opportunity to make corrections for multiple comparisons (familywise error rate, FWER). Since the research is exploratory, we carried out an analysis of the long-term area of the action, without breaking it up into separate periods of analysis. This raises the threshold for familywise error correction (FWEC), but the results are more resistant to accepting false alarms. In the statistical analysis, an interval from −1.5 to 0.5 s relative to the time of the maximum throw speed was used, and it included all phases of the throw: raising the hand, backward move, acceleration, throwing and follow-through.

A repeated measurement ANOVA, involving four throw categories (1PP, 3PP0, 3PP20 and 3PP40), was used to analyze the effects. The significance level was calculated using permutation statistics; the number of permutations was 1000. The FWER adjustment was used to account for multiple comparisons. Paired differences were evaluated using Student’s *t*-test (with FWER correction). Regression analysis was used to study the prediction of throw accuracy based on kinematics data.

The significance level was *p* < 0.05.

## 3. Results

### 3.1. Accuracy Analysis

Analysis of accuracy was carried out on the data of number of throws and mean hit accuracy in each condition.

#### 3.1.1. Target Hit Accuracy

One-way ANOVA of the number of successful hits reveal the significant effect of Condition (F(3,81) = 30.77, *p* < 0.001, *ε* = 0.961, $\eta_{p}^{2}$ = 0.533). The number of hits gradually decreases (Table 1) from 1PP to 3PP0 and 3PP20, but 3PP20 does not differ from 3PP40 (Table 2). Therefore, the number of successful hits is affected by perspective distortion and angle distortion but does not depend on the angle value.

#### 3.1.2. Hit Accuracy

**Figure 3:**
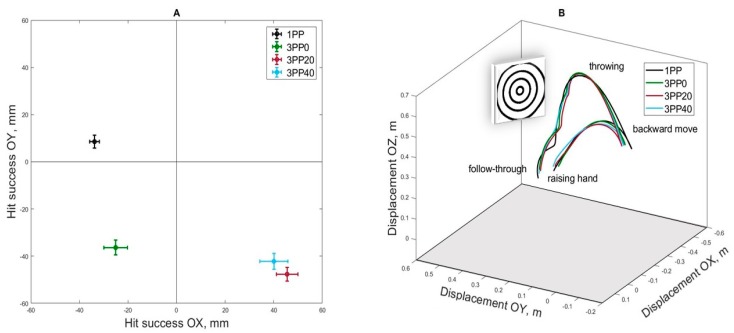
Hit accuracy and hand trajectories during the dart throws. (**A**) Mean hit success in the 1PP, 3PP0, 3PP20 and 3PP40 conditions along the OY and OZ axes and the standard error of the mean (SEM). (**B**) Mean trajectories of the right hand during a throw for four conditions. 1PP—first-person condition, 3PP0—3PPs with 0°, 3PP20—3PP with 20°, 3PP40—3PP with 40°.

The accuracy of hits of each category is presented in Figure 3 and Table 1. Firstly, we conducted the analysis on differences in mean hit accuracy between conditions. ANOVA was calculated with the factors condition (1PP, 3PP0, 3PP20 and 3PP40) and axis (OX and OY). Results revealed significant effects of condition (F(3,81) = 8.30, *p* < 0.001, *ε* = 0.773, $\eta_{p}^{2}$ = 0.235), axis (F(1,27) = 24.10, *p* < 0.001, *ε* = 1.000, $\eta_{p}^{2}$ = 0.472) and interaction condition × axis (F(1,27) = 35.88, *p* < 0.001, *ε* = 0.781, $\eta_{p}^{2}$ = 0.571). Separate ANOVA calculated for each axis revealed a significant effect of condition in OX axis (F(3,81) = 25.20, *p* < 0.001, *ε* = 0.771, $\eta_{p}^{2}$ = 0.483), pair comparison showed that 1PP and 3PP0 differed from 3PP20 and 3PP40 (*p* < 0.001, Bonferroni test). The effect of condition in OY axis was also significant (F(3,81) = 19.44, *p* < 0.001, *ε* = 0.841, $\eta_{p}^{2}$ = 0.419) and pair comparison revealed that 1PP differed from all 3PP conditions (*p* < 0.001, Bonferroni test). 

A statistical analysis of pairwise differences in the number of successful hits, average absolute accuracy values along the OX and OY axes and equality of variances of successful hits along the OX and OY axes are presented in Table 2.

Additionally, we analyzed the deviation hit accuracy from the OX and OY axes using one-sample *t*-tests. The results indicate that conditions with a natural perspective angle (1PP and 3PP0) show a significant bias for hitting to the left side of the target and conditions with a changed perspective angle (3PP20 and 3PP40) shows a significant bias for hitting to the right side of the target (Table 1). The absolute accuracy of hits along the OY axis showed that in the 1PP the deviation was non-significant and in 3PP conditions hits were in the lower part of the dartboard.

Thus, the absolute accuracy along the OX axis was affected by the parameter of distortion of degree and the absolute accuracy along the OY axis was affected by the parameter of distortion of perspective.

Secondly, we analyzed the relative differences between the conditions. 

The relative accuracy along the axis OX revealed the statistically significant difference in all pairs, associated with 1PP, and did not reveal a statistically significant difference in all other pairs. Relative accuracy along the OY axis did not reveal statistically significant differences in all pairs of conditions.

Therefore, change in perspective from 1PP to 3PP resulted in a decrease in the total number of successful hits, absolute accuracy along the vertical axis towards the bottom of the dartboard, and a decrease in relative accuracy along the horizontal axis. Change in the angle of perception in 3PP resulted in additional decrease in the total number of successful hits, and absolute accuracy along the horizontal axis was shifted towards the right side of the dartboard. In other words, we had 3 groups (1PP, 3PP0 and 3PP20 with 3PP40) that are statistically significantly different in terms of the number of successful throws (*N*) and the absolute accuracy of hits along the OX and OY axes. These conditions can be designated as a condition with a natural perspective and a natural angle (1PP), a condition with a distorted perspective and a natural angle (3PP0) and a condition with a distorted perspective and a distorted angle (3PP20 and 3PP40).

Control analysis was performed to investigate the sex differences in the number of hits and mean accuracy. Sex differences in the number of hits reveal the main effect of Sex (F(1,26) = 7.37, *p* = 0.012, $\eta_{p}^{2}$ = 0.221), indicated that males have more hits (31.7 ± 0.80) than females (27.9 ± 0.97). The interaction sex × condition was non-significant (F(3,78) = 1.70, *p* = 0.173, $\eta_{p}^{2}$ = 0.061). The main effect of sex in hit success was non-significant for both OX and OY axes (OX: F(1,26) = 0.83, *p* = 0.371, $\eta_{p}^{2}$ = 0.031; OY: F(1,26) = 2.38, *p* = 0.135, $\eta_{p}^{2}$ = 0.084). The interaction sex × condition was also non-significant (OX: F(3,78) = 0.43, *p* = 0.729, $\eta_{p}^{2}$ = 0.016; OY: F(3,78) = 0.39, *p* = 0.757, $\eta_{p}^{2}$ = 0.015).

Power analysis with the goal power equal to 0.9 and the alpha equal to 0.05 for the given total standard deviation and condition’s means reveal that for the number of hits required 12 subjects, for the hit success along the OX axis required 11 subjects and for the hit success along the OY axis required 13 subjects. This is more than two times less than in the current study.

### 3.2. Kinematic Analysis

The analysis of kinematic data was carried out for the successful throws in each condition using the displacement, velocity and acceleration of the right hand as well as rotation angle of the torso and displacement angle in the period of a throw. Figure 3B shows the trajectories of the right hand movement (positioned in three-dimensional space) when darts were thrown from the 1PP and the 3PP with 0°, 20° and 40° angles of perspective distortion. The mean values of hand displacement significantly differed when the perspective changed, the path of hand displacement become less amplitude along the axis of the throw in backward move period and less downward move in the follow-through period, relative to the normal 1PP. The displacement data along the OX axis did not produce statistically significant effects and were not reported.

#### 3.2.1. OY axis

##### Displacement

**Figure 4:**
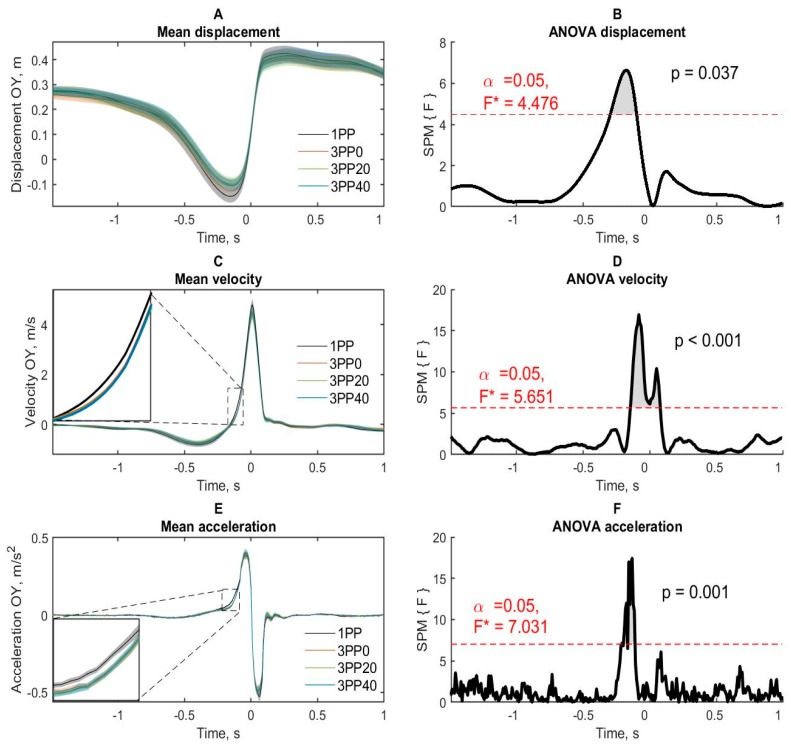
The right-hand kinematic data along the OY axis. (**A**,**C**,**E**) Displacement, velocity and acceleration of the right hand in four conditions of throws. Semi-transparent areas around lines are standard errors of the mean. (**B**,**D**,**F**) ANOVA effects of the condition. Red dashed lines denote the statistically significant level (with familywise error rate (FWER) correction). 1PP—first-person condition, 3PP0—third-person perspectives with 0°, 3PP20—third-person perspective with 20° and 3PP40—third-person perspective with 40°. Time 0 represents the moment of peak velocity of the right hand moving along the OY axis (along the direction of the throw).

The throwing condition (1PP, 3PP0, 3PP20 and 3PP40) produced a significant effect on the hand movement (Figure 4A) in the period from −0.298 to −0.099 s (Figure 4B).

Pairwise comparisons showed (Supplementary Materials Figure S4) that right hand displacement was more negative in 1PP in comparison with 3PP20 (from −0.265 to −0.116 s) and 3PP40 (from −0.257 to −0.158 s) conditions. There were also almost significant differences in movement in the similar period in 3PP0 condition compared to 1PP condition. The 3PP conditions were not differ from each other.

##### Velocity

Significant effects of the throw conditions (Figure 4C) in the periods from −0.149 to 0.074 s were obtained (Figure 4D). Pairwise comparisons showed that the velocity in 1PP condition was higher compared to 3PP0 (from −0.133 to −0.066 s and from 0.041 s to 0.066 s), 3PP20 (from −0.133 to 0.075 s) and 3PP40 (from −0.142 to −0.05 s) conditions. The 3PP conditions did not differ from each other.

##### Acceleration

The significant effects of the throw conditions (Figure 4E) in the periods from −0.217 to −0.108 s were obtained (Figure 4F). Pairwise comparisons showed that during the period of backward move and retraction of the right hand, the hand acceleration in 1PP condition was higher compared to 3PP0 (from −0.167 to −0.125 s), 3PP20 (from −0.192 to −0.117 s) and 3PP40 (from −0.192 to −0.125 s) conditions. The 3PP conditions did not differ from each other.

These results indicate that the hand kinematics in the 1PP condition had lower amplitude and was smoother in the period from the backward move to the time after the peak velocity.

#### 3.2.2. OZ axis 

##### Displacement

**Figure 5:**
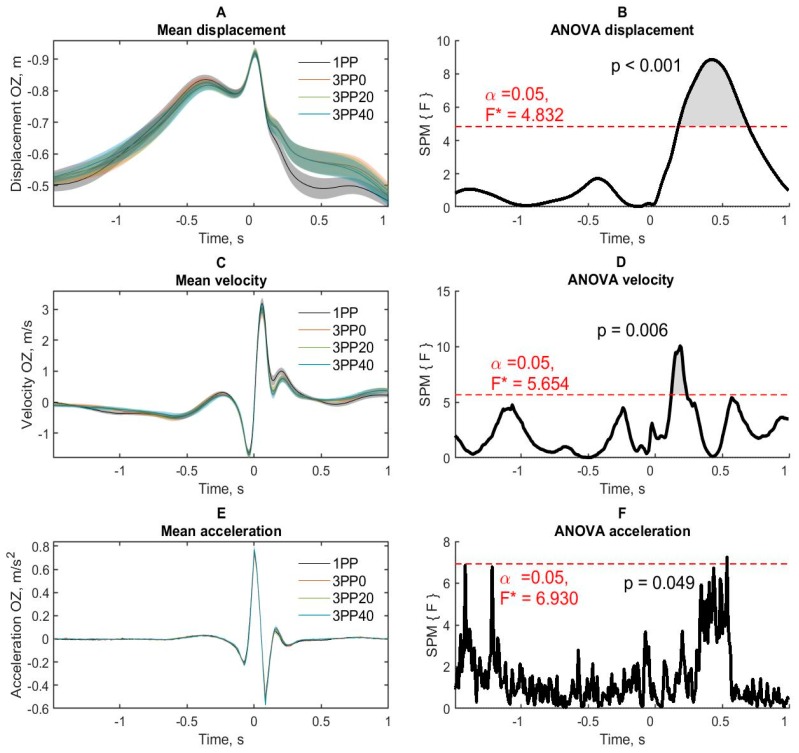
The right hand kinematic data along the OZ axis. (**A**,**C**,**E**) Displacement, velocity and acceleration of the right hand in four conditions of throws. Semi-transparent areas around lines are standard error of mean. (**B**,**D**,**F**) ANOVA effects of the condition. Red dashed lines denote the statistically significant level (with FWER correction). 1PP—first person condition, 3PP0—third-person perspectives with 0°, 3PP20—third-person perspective with 20° and 3PP40—third-person perspective with 40°. Time 0 represents the moment of peak velocity of the right hand moving along the OY axis (along the direction of the throw).

Significant effects of the right hand displacement were found in periods from 0.166 to 0.689 s (Figure 5A,B).

Pairwise comparisons showed that the hand displacement was higher in the 1PP condition as compared to 3PP0 condition (from 0.174 to 0.697 s), 3PP20 condition (from 0.216 to 0.647 s) and 3PP40 condition (from 0.348 to 0.539 s). The 3PP conditions did not differ from each other.

##### Velocity

The significant effects of the throw conditions (Figure 5C) in the periods from 0.116 to 0.233 s were obtained (Figure 5D). Pairwise comparisons showed that during the period of lowering the hand down after throwing a dart, the hand velocity in 1PP condition was higher compared to 3PP0 (from 0.142 to 0.208 s), 3PP20 (from 0.166 to 0.258 s) and 3PP40 (in the period from 0.133 to 0.192 s) conditions. The 3PP conditions did not differ from each other. 

##### Acceleration

Acceleration of right hand movement had non-significant effects of throwing conditions (Figure 5E,F). 

Thus, the subjects lowered their right hand after the throw with the greater amplitude and velocity in the 1PP compared to the conditions with the distorted perspective.

#### 3.2.3. Torso Rotation

**Figure 6:**
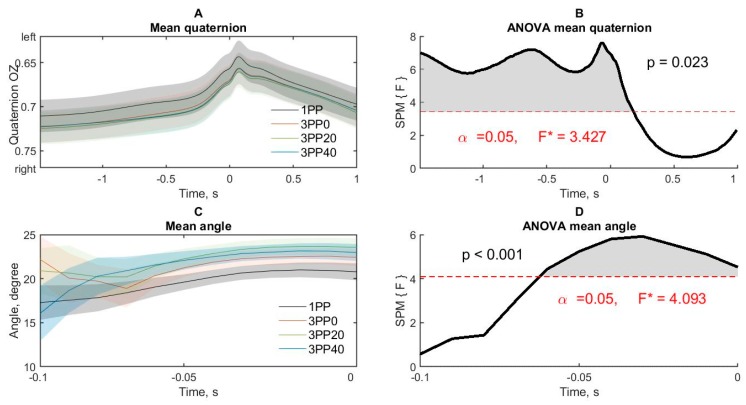
The angle data of torso rotation and hand movement during the dart throwing. (**A**) Torso rotation angle on the OZ axis in four conditions of throws. Semi-transparent areas around lines are standard error of mean. (**B**) ANOVA effect of the condition on the rotation angle of the torso. Red dashed lines mark the statistically significant level (with FWER correction). (**C**) Direction of the right hand movement in four conditions of throws. (**D**) ANOVA effect of the condition on degree of direction of movement of the right hand. Red dashed lines mark the statistically significant level (with FWER correction). 1PP—first person condition, 3PP0—third-person perspectives with 0°, 3PP20—third-person perspective with 20° and 3PP40—third-person perspective with 40°. Time 0 represents the moment of peak velocity of the right hand moving along the OY axis (along the direction of the throw).

To study the rotation of the body during a throw, a torso quaternion around the OZ axis was used. The significant effects of the throw conditions (Figure 6A) in the periods from −1.5 to 0.183 s (Figure 6B) were found.

Pairwise comparisons showed that the subject’s torso was turned more leftward (counterclockwise) during periods of raising the arm, moving backward and throwing in the 1PP condition compared to 3PP0 (from −1.5 to −1.283 s and in the period from −0.1 to 0.067 s), 3PP20 (from −1.5 to 0.075 s) and 3PP40 (from −1.5 to 0.05 s) conditions. Thus, the throw in the 1PP was accompanied by a more natural torso rotation counterclockwise, which makes it more convenient to position the right hand concerning the target.

#### 3.2.4. The Movement Angle during a Throw

Dart accuracy significantly depends on the hand position in space during dart release time [18], and the dart release occurs several milliseconds before the peak velocity [37]. So, the direction of the hand movement in a short period immediately before and in time of the peak velocity includes the dart release time and should affect hit accuracy. In the present experiment, the hand movement direction was represented as the angle between the direction of motion and the OX axis in the XY plane.

The significant effects of the throw conditions (Figure 6B) in the periods from −0.058 to 0 s were obtained (Figure 6D). Pairwise comparisons showed that at the time of throwing the dart, the angle of the direction of hand movement was significantly greater in the 3PP condition at 20 degrees (from −0.058 to 0) and 40 degrees (from −0.05 to −0.008 s) relative to the 1PP condition. 

Thus, the change from the first to the 3PP results in shifting the torso direction to the right and increasing in the angle of hand movement immediately before and at a time of dart release.

### 3.3. Effect of Kinematic on Accuracy

It was previously described that conditions affect the absolute accuracy along the OX axis, however, they did not affect the hand movement. In this section we presented the results of regression analysis, predicting hit accuracy from kinematic data.

The regression analysis did not reveal statistically significant effects of displacement, speed, acceleration along the OX and OY axes and torso rotation angle on the absolute accuracy of hitting along the OX and OY axes. However, an angle of the movement during the throws predicted the absolute accuracy along the horizontal axis (F = 2.38, *p* = 0.049) in 1PP conditions at time −0.050 s.

Further, we analyzed the relative data. The analysis included the differences between 3PP20 and 1PP as well as between 3PP40 and 1PP in hand movement angle and accuracy gain along the OX axis between the corresponding conditions since these variables had significant differences between conditions. The angle data differed significantly from the normal distribution, so we used a non-parametric Spearman correlation. The analysis was carried out in 12 points before the time of peak velocity, i.e., from −0.100 to 0 ms. For the difference between 3PP20 and 1PP it was found significant correlations between angle difference and hit gain from −0.042 to 0 c (*p* < 0.048) with maximum correlation is −0.017 c (R = 0.57, *p* = 0.002). For the difference between 3PP40 and 1PP it was found significant correlations between angle difference and hit gain from −0.083 to 0 c (*p* < 0.049) with maximum correlation is −0.050 c (R = 0.66, *p* < 0.001).

Power analysis calculated for the point of maximum effect of movement angle with the goal power equal to 0.9 and the alpha equal to 0.05 revealed that for the given total standard deviation and condition’s means the study required six subjects. The required sample sizes for other variables were greater than in the present study.

## 4. Discussion

The present study revealed the changes in accuracy and hand kinematic while the subjects performed dart throws perceiving themselves from 1PP and from the 3PP with various degrees of perception. The results of the experiment showed that in the 1PP condition the number of successful hits decreased and the mean accuracy of the hits shifted downward compared with the 3PP conditions. Shifting the angle of self-perception in the 3PP rightward results in shifting mean accuracy in the same direction. Kinematic analysis revealed that in the 1PP condition displacement speed and acceleration of the right hand in the direction to the target in the period from a backward move to the time of maximum velocity were greater compared with 3PP. In the follow-through period amplitude and velocity of the lowering the hand was greater in the 1PP condition compared with 3PP condition. Finally, the torso angle was greater in the 1PP condition compared with 3PP condition and hand movement angle before the time of peak velocity was smaller in the 1PP condition compared with 20 and 40 degrees of the 3PP condition.

The motor program essentially depends on the visual feedback—reafferentation [7], which in turn is associated with the perception of acting subject’s localization. Studies [17,18,32] showed that the subject’s own actions affect the effectiveness of mental rotation in the way of reducing interference in the perception of subject’s hand from 1PP. Another study suggests that changing the perspective reduces the speed and accuracy of movements [4], which was confirmed by the results of this study. The angle of a virtual avatar [28] and one’s own body [9] influence perception accuracy when changing the perspective of perception—the congruent position of the stimulus or avatar compared to the position of the subject’s own body facilitates perception.

In the present study, subjects performed dart throws from the 1PP in a situation of congruence of visual and proprioceptive afferentation, and from a 3PP in a situation of conflict between visual and proprioceptive information. The amount of conflict was increased by increasing the angle of the self-perception from the 3PP from 0 to 20 and 40 degrees.

The number of successful hits decreased gradually from 1PP to 3PP0 and to 3PP20 and stabilized at 40 degrees. So, changing the angle from 1PP condition to 3PP0 condition was about 5 degrees above the view. According to data [33,34] the border for automatic movement regulation was 14–15 degrees. In the present experiment shifting the angle from 1PP to 3PP with zero degrees was under this border. The angle of view was also not shifted laterally, Level 1 of regulation [8,9,10] should not be affected and the target was clearly visible in front of subject view. However, change in perspective of view in the present experimental results in a decrease in movement success. These results, in according with similar reports [5,6], showed a decrease in accuracy in 3PP condition. However, in report by Watanabe R. and Higuchi [6] was angle rotation of 180 degrees, but in the report by Gorisse et al., [5] was zero degrees’ rotation, so, it is unclear the relative contribution of the 3PP condition and rotation angle. The results of the current study shed light on this topic. Particularly, increase the lateral angle in the 3PP from 0 to 20 and 40 degrees induced an additional decrease in the number of hits. The source of this decrease lay in controlled motor regulation i.e., angle changed with extend exceeding the border of controlled regulation [33,34] and should involve the Level 2 regulation, which is associated with the limited capacity of the working memory [8].

Shifting in perspective from 1PP to 3PP0 results in shifting the mean accuracy downward, i.e., in the opposite direction. However, shifting the perspective angle rightward from 3PP0 to 3PP20 and 3PP40 caused shifting the mean accuracy also in rightward, i.e., in the same direction. Thus, in the present study was found the dissociation between the accuracy along horizontal and vertical axes. Orthogonal changes assume different mechanisms. Vertical axis associated with overcoming gravity, thus, requires muscle effort. Throwing to the greater distance required the greater muscle effort for giving more impetus for higher and longer flying of the object. The effect of the third perspective, but not the horizontal angle, expressed in reducing the hit’s relative accuracy. Changes in muscle effort could be produced by the illusory change in the estimation of the distance and self-body height. In the present experiment, the location of the camera was backward and higher than the participant’s eyes position and viewing from this reference frame may evoke the illusion. Alternatively, perceiving one self’s body may increase attention to the movement or inner focus of the attention [40]. It is also known that the inner focus of attention decreases the accuracy in dart hitting [41].

Changes in the horizontal axis required only a shift in movement accuracy. However, this does not explain why the mean accuracy of the hits shifted in the congruent direction with the perspective angle. A possible reason for this may lay in the perception of target location. When the perspective is shifted to the right in the 3PP, the subjects perceive the target in between the self-body and actual viewpoint, i.e., illusory shifted rightward. Implying that the motor program also shifts the direction of the movement rightward. 

Changing the angle of view is studied with the help of lenses and results in the gradual recovery of motor regulation [18]. Changing the perspective also has a negative effect on the movement accuracy [5,9,10,28]. In the current experiment, we found that perspective and angle have a different impact on the hit accuracy of the dart throwing. Changing perspective by viewing oneself from back and up shifts the reference frame, resulting in a decreased number of hits, decreased horizontal relative accuracy, shifting the mean accuracy of the dart-throwing downward due to the changing the estimation of distance, and self-body height or evoked inner focus of attention. Shifting the angle additionally evoked the controlled mental rotation mechanism with limited working memory capacity results in an additional decrease in the number of hits and shifting mean accuracy in congruent direction due to the illusory shifting the target location. 

The accuracy data in the current study are complemented by the kinematic results. Data of [5] revealed the decrease in velocity of actions in the 3PP. In the current study, the analysis of the trajectory and velocity of the right hand suggested that subjects perform hand movements in 3PP condition with lower amplitude during the backward move period and lower speed and acceleration immediately before the peak velocity. These data are consistent with the action constraint hypothesis [42] because the subjects were seeing their own hand in the 3PP condition, their inner focus of attention [40] was increased, and they tried to reduce the conflict between the perception of their hand from the outside and the need to complete the motor program, which also attracted attention to the process of executing the movement.

The difference between the 1PP and 3PP conditions in terms of displacement, speed, and acceleration significantly overlap in time and are located in the period −200 to −100 ms. In studies [29,31,43], it was found that motion correction when changing the location of the target during movement occurs after 115–147 ms due to a mismatch of proprioceptive data with the forward model. However, unlike previous studies using a change in the position of the target during the movement of the hand, in the present study the position of the target remained constant, only the angle of perception of oneself and the target are changed. Therefore, we assumed that the visual control of movement when perceiving oneself from a third-person perspective is increased due to a mismatch between visual and proprioceptive perception. Higher control of movement in turn causes a decrease in the amplitude and dynamic characteristics of its implementation. In other words, approximately 100–200 ms before the release of the dart, subjects try to control the hand to resolve the conflict between the visual and proprioceptive systems. The data are consistent with the results of [44], which showed that the accuracy of the throws is positively related to the peak of acceleration of the hand, activity of posterior deltoid muscle and negatively related to activity of thenar muscle.

Changes in the amplitude and dynamic characteristics of movement in third-person perspective condition can also be caused by changes in the estimate of the distance to the target and the perception of the height of one’s own body. Upon an illusory change in the distance [32], independent contributions of the perceptual and motor components to eye movement patterns were found.

A decrease in the amplitude and speed of the throwing hand is also accompanied by a shift of hits downward, however, a decrease in the position of the hand along the OZ axis before the peak velocity does not occur. A downward shift in the accuracy can be associated with a lower speed of the hand that transmits the impulse to the object. Indeed, the speed of the hand in the target’s direction was less from the 3PP compared to the 1PP. This, in turn, can be caused by attention to the hand in the condition of performing throws from the third-person perspective. The data [41] indicate that with inner focus of attention to the limb, the muscle activity of the triceps brachii agonist increases when darts are thrown. In a study [45], attention to a limb increases the tension of antagonistic muscles and reduces the effectiveness of the regulation of movement.

Further hand movement becomes faster, and subjects did not have time to perform the movement correction based on the real perceptual feedback [30], so the hand movement executed based on the inverse model and predicted proprioceptive feedback [25]. Thus, amplitude and speed characteristics of the hand movement in the dart release time did not differ between conditions in the current experiment, except velocity along OY axis. Effect of peak velocity at this axis was small and was accompanied by two greater effects before and after peak velocity, i.e., during the hand acceleration and hand slowing.

The second period of differences in hand velocity along OY axis could be induced by two causes. First, in the 1PP condition, the hand moved on a ballistic trajectory with the greater speed and the subject had to make more effort to stop it. In the 1PP condition the hand movement amplitude was greater in the period of the backward move and also tendency to increase in amplitude following dart release (Figure 4A). Contrastingly, in the 3PP condition, slower hand movement during the dart throwing could result in better hand control and early stopping of the hand. Additionally, reducing the speed of the hand movement in the 3PP condition is evoked by the conflict between visual and proprioceptive information. Perhaps, both hypotheses are true and the first is more applicable for the explanation of the hand movement from the 1PP and the second explains better the movement regulation from the third-person perspective. 

Following the dart release, subject’s attention is directed to assessing the throw accuracy and drooping hand no longer carries the functional significance for dart throw. However, in the current experiment, we found smaller hand amplitude and velocity along the vertical axis in 3PP condition. A similar effect was found in our previous study [36] in the eyes-closed condition. In the current experiment in the follow-through period subject’s attention was rather directed to target for assessing the throw accuracy, than to the fallen hand. Dart flying time plus time of visual and cognitive analysis exceeded the time when differences between hand velocity in the first and 3PP conditions were started. So, the differences between conditions in amplitude and velocity were not evoked by assessing the throwing accuracy but associated with the motor conflict. To the 100–200 ms follow the peak velocity the hand movement occurred with less velocity (Figure 4C) and motor regulation based on proprioceptive feedback was available. So, the conflict between visual and proprioceptive data become actual again in a condition such as less relaxed biceps muscle and less lowered hand.

As an initial hypothesis of our study, we suppose that shifting the viewpoint in 20 and 40 degrees induced shifting the hand movement in the same direction in order to combine the eye, hand and target in line. However, in this study, we did not find differences in movement, speed or acceleration in the direction of the shift in the angle of perception. The absence of differences along the OX axis could be caused by the fact that, for hitting a relatively small target (10°50′), sufficiently strong restrictions are imposed on the movements to ensure accuracy of hits. Considering that the majority of misses in the third-person perspective conditions were to the right from the target, it can be assumed that a larger target size would catch a larger rightward deviation of hitting caused by stronger deviations of the arm moving in the same direction.

In order to identify the shifting of the body position as a whole and the hand in particular, with an increase in the third-person condition angle, we performed an additional analysis of the subject’s body rotation and the hand angle in the XY plane during the throws. We found that in the 1PP condition, the body was turned more leftward, that is, counterclockwise, in the period from −1.5 to 0.183 s and, therefore, the right shoulder and hand were closer to the virtual line connecting the subject’s eye and the target. This position most closely matches the ability to control the throw. The perception of oneself behind the third-person, regardless of the angle, leads to a smaller turn of the body leftward and the shoulder becomes more perpendicular to the line of the throw. A smaller turn of the body to the left when performing third-person perspective throws imposes physical limitations on the possibility of motor regulation. It should also be noted that the differences are not related to the different settings of the subjects during the throws. As indicated in the method section, all subjects stood on marks on the floor and their leg position was the same for all subjects in all conditions. Consequently, the differences found concern torso rotation caused by cognitive factors, but not the subjects’ physical position.

Since the angle of torso’s rotation was greater in the first-person condition compared with the third-person condition from the very beginning of the analysis period, we assumed that the rotation of the torso was associated with proactive motor control and the motor setting, when the movement was performed. It should also be noted that the time of differences between the angles of torso’s rotation from the first and 3PPs ends 183 ms after the time of the hand’s velocity peak, which corresponds to the time of motion compensation when changing the target’s localization [9,33,38]. The influence of the cognitive factor can be traced in the fact that significant differences between the conditions of the first and 3PPs in terms of the angle of torso’s rotation end at about the same time period when the amplitude differences of lowering the arm begins. This suggests that proactive motor control ends after the completion of the throw, but the cognitive conflict caused by third-person perception continues to be pronounced and affects the amplitude of movements.

Finally, according to the angle of the hand movement in the XY plane, it was found that the hand moves from right to front. A smaller value of the angle indicates a greater deviation of the hand movement to the right. The results showed a smaller angle of the hand movement in the 1PP condition (Figure 6C), that is, the hand movement was directed more from right to front (Supplementary Materials Figure S1A). In the third-person perspective condition with a viewing angle of 20 and 40 degrees, the hand moved with a larger angle relative to the OX axis, that is, more straightly towards the target. Differences in direction reach a significant level immediately before and at the time of the peak velocity and overlap in time with differences in speed along the OY axis. These results indicate that the change in the angle of perception in the third-person condition affects the program of movement, which deviates in the opposite direction. These results also correlate with the change in the average accuracy of dart throws. A larger angle of the hand movement’s direction from right to forward in the 1PP condition is reflected in the displacement of hits to the left, relative to the direction of hand movement, and a straighter movement of the hand towards the target in 20 and 40 degrees of the third-person perspective conditions is reflected in the displacement of hits to the right. Thus, a change in the angle of perception to the right causes the opposite change in the direction of hand movement, which in turn predicts a shift in the average accuracy of hits, congruent to the shift in the angle of perception. Moreover, the angle of rotation of the torso does not correlate with the displacement of the hit. These results correspond to the data [37,46], which showed the existence of independent parameters of the place and the time of release of the dart, affecting the hit’s accuracy. According to [37], the moment of dart release occurs before the time of the peak velocity for 2–11 ms, that is, in the results of this study, the deviation of the hand movement occurs at the time of the release of the dart.

Oppositely directed changes in the angle of perception and the angle of hand movement could be caused by the illusion of a target’s displacement. While the subject illusory was seeing the target on the right, to shift the desired hit in the same direction, the subject needed to move his hand until the moment of peak velocity with a lesser shift to the right. Probably, the reference frame was also shifted to the point where the dart was released, so the less the subject was prone to illusion, the greater was the deviation of the hand from the axis of forward movement and the smaller was the deviation of hitting to the right in the third-person condition, compared to the third-person condition. However, a larger increase in the angle of perception did not lead to a larger increase in the angle of hand movement.

Regarding the angle of the perception one could think, that current results were similar to that used the lenses for visually shifting the position of the target [18,30]. However, the subject in these experimental conditions localizes itself in the same place and corrects the movement of the hand under the changed angle of view in order to match the egocentric position. In the third person view condition in the current experiment, the subject locates himself in a different place and adopts the hand movement to a changed perception of the self-location. Thus, the movements are adapted not only in accordance with the sensory flow but also in accordance with the subject’s location.

Thus, decreasing the hand amplitude and speed in the 3PP condition could be induced by the conflict between proprioceptive and visual information. As a result, attention is shifted from controlling the accuracy of the throw to the movement regulation. Studies have shown that the inner focus of attention, controls the bodily implementation of the action, reduces the accuracy of the action. In opposite, the external focus of attention, aimed at controlling an object, increases the accuracy of dart throws and reduces muscle tension [41]. In the present study, 3PP condition evoked proactive motor regulation, reflected in less torso rotation. Then the throws were executed with less amplitude of backward move, less velocity and acceleration of the hand before dart release, close to the time of maximum velocity, as well as, less amplitude and speed of hand lowering follow the dart release. These periods were more than 100 ms before and after time of peak velocity and associated with the movement regulation based on both visual and proprioceptive feedback [29,31,43], which is in the conflict in the 3PP condition in the current study. In the period around the peak speed, the hand movements were too fast to be regulated based on the real feedback information. So, the hand movement in a short period around dart release was not affected by the third-person perspective and realized based on both the inverse model and predicted sensory information of the forward model [25]. On this period shifting angle of the movement predicts a rightward shift in the mean accuracy. Since the inverse model was not trained for the movement regulation in changed perceptual condition, the hand kinematic and throw accuracy executed with low prediction error correction and changed significantly. Finally, follow the dart release the influence of proactive motor control disappeared. 

The effect of changing the perception angle by 20° and 40° to the right on the dart throwing results can manifest itself in a similar way with dysfunction of somato-spatial synthesis while correlating the object’s location with the respect to one’s body and constructing a corresponding action program. For example, a mismatch of visual and proprioceptive information is observed in patients with optic ataxia associated with a dysfunction of the parieto-occipital junction [47]. Conducting cognitive diagnostics in VR or cognitive rehabilitation in VR by using Vicariation or by restructuring functional systems, without taking into account the factor of perception distortion of the perspective and body proportions, can produce an inadequate assessment on the degree of cognitive dysfunction. Studies have also shown that out-of-body experience [13] and body schema [20,21] are associated with the activity of the right inferior parietal cortex. The perception of oneself from the 3PP in the current study may be due to the activation of these areas of the cortex.

The practical application of the results of this study also may extend to other areas. In the gaming industry, the perception and management of an avatar require a change in the perception perspective. In some areas of industry, there are requirements of online control of robotic actions, based on the operator’s movements. In sports or dancing, training often takes place in a hall with mirrors, and the regulation of movements is based on a mirror reflection, that is, from the third-person view.

The present study had several limitations.

Firstly, there were technical limitations of the cameras used. HTC VIVE Pro HMD built-in cameras had poor image quality and USB-camera used to simulate third-person perspective provided 2D images. This imposes restrictions on the assessment of the accuracy of hits by the subjects. There was also a slight but noticeable hardware delay when transmitting images from a first-person view camera and a third-person view camera.

Secondly, changes in the kinematics of motion associated with the changes in perspectives can affect different links of the motor chain. Therefore, in addition to analyzing the rotation of the torso and the angle of the direction of movement of the hand, the analysis of the angles and position of the shoulder, arm and forearm will provide additional information and will help to better understand the motor effects of changing perceptual perspectives. 

Thirdly, we had not registered the time of dart release and dart flight trajectory. Since the time of dart release and time of the hand peak velocity are close to each other [37], we propose that selection of peak velocity as a reference point reflect the inner nature of this movement, aimed to speed up the dart. However, third-person perspective condition could change the timing between dart release and peak speed.

Fourthly, target height was located on a fixed position for all subjects. Since the height of the subjects was varied, the target was located above the eyes for some subjects and below the eye for other subjects. These differences could affect the hand kinematic. In future research, it would be preferable to set the height of the target at an individual level.

Fifthly, although there were enough participants to make conclusions, but for kinematic, except hand movement angle, G power analysis showed that the number of participants was insufficient. 

Finally, in the current experiment, some error throws were outside the target area. In most cases these throws in the 3PP conditions were shifted rightward. So, it seems justified to use the target with larger area in order to catch the throws with large deviation from the center of the target.

## 5. Conclusions

In the present study, we investigated the change in accuracy as well as hand and torso movement when making dart throws in the first and third-person perspectives with varying angles. The results indicated that the mean accuracy shifted in the opposite direction in the vertical axis and congruent direction in the horizontal axis. Kinematic data revealed less torso angle in the 3PP conditions before and during the throw. The amplitude, velocity and acceleration in 3PP condition were decreased as compared with 1PP condition before and after the peak velocity and the same variables did not differ between conditions more than 100 ms around peak velocity period. The hand movement angle toward the target direction was smaller in the 3PP conditions with 20 and 40 degrees compared with the 1PP condition just preceding the time of peak velocity and the difference between conditions predicted the changes in mean accuracy of the throws. Thus, the results of the current study revealed that the transformation of the motor program takes into account the subject’s localization.

## Figures and Tables

**Table 1:** The number of throws and the Mean (SEM) of the hit success along the OX and OY axes in 1PP (1PP) and 3PPs with 0°, 20° and 40° (3PP0, 3PP20 and 3PP40).

	1PP	3PP0	3PP20	3PP40
Number, *N* (SEM)	36.0 (0.72)	30.3 (1.06)	27.3 (1.19)	25.3 (1.13)
Success OX (SEM), mm	−33.8 (4.01)	−25.1 (9.8)	45.4 (8.86)	40.1 (11.43)
Success OY (SEM), mm	8.5 (5.51)	−36.3 (6.31)	−47.6 (5.76)	−42.2 (6.81)
One-Sample *t*-Test, OX (tstat, *p*-value)	−8.293 (<0.001)	−2.496 (0.018)	5.038 (<0.001)	3.438 (0.001)
One-Sample *t*-Test, OY (tstat, *p*-value)	1.517 (0.140)	−5.659 (<0.001)	−8.125 (<0.001)	−6.082 (<0.001)

**Table 2:** Statistical effects of differences in the number of successful hits, absolute and relative accuracy of hits.

	Stat-Value (*p*-Value)	1PP vs. 3PP0	1PP vs. 3PP20	1PP vs. 3PP40	3PP0 vs. 3PP20	3PP0 vs. 3PP40	3PP20 vs. 3PP40
Number of successful hits	Paired-sample *t*-test, *N*	5.136 (<0.001)	8.181 (<0.001)	9.148 (<0.001)	2.377 (0.024)	3.825 (<0.001)	1.635 (0.113)
Absolute hit accuracy	Paired-sample *t*-test, OX	−0.794 (0.433)	−7.669 (<0.001)	−5.898 (<0.001)	−5.814 (<0.001)	−4.345 (<0.001)	0.609 (0.547)
	Paired-sample *t*-test, OY	6.235 (<0.001)	6.794 (<0.001)	5.117 (<0.001)	1.298 (0.205)	0.779 (0.442)	−0.717 (0.479)
Relative hit accuracy	Two-sample F-test, OX	6.097 (<0.001)	4.901 (<0.001)	8.197 (<0.001)	0.805 (0.578)	1.340 (0.452)	1.664 (0.193)
	Two-sample F-test, OY	1.309 (0.490)	1.090 (0.823)	1.526 (0.278)	0.834 (0.639)	1.167 (0.692)	1.400 (0.388)

## Supplementary Materials

The following are available online at https://www.mdpi.com/2076-3425/10/1/55/s1.
