# Supplementary material for: Motor Program Transformation of Throwing Dart from the Third-Person Perspective
Source: Brain Sci. 2020 Jan 18;10(1):55. doi: 10.3390/brainsci10010055 (PMC7016666; doi:10.3390/brainsci10010055)
Supplement: Supplementary file 1 [file brainsci-10-00055-s001.pdf]

Supplementary Material: Motor Program Transformation of Throwing Dart from the Third-Person Perspective

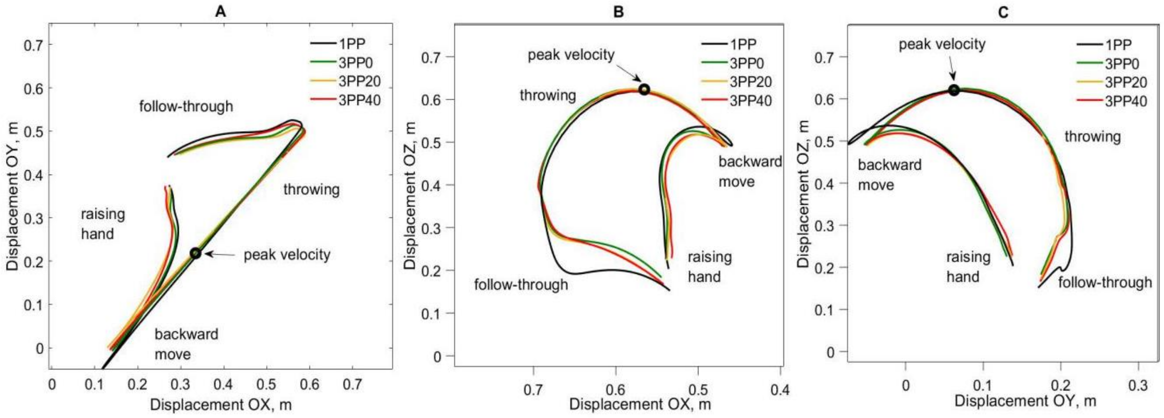

Figure S1. Mean right hand trajectories when throwing darts in the four Conditions for all subjects. **A.** The trajectory of the right hand on the XY plane. **B.** The trajectory of the right hand on the XZ plane. **C.** The trajectory of the right hand on the YZ plane.

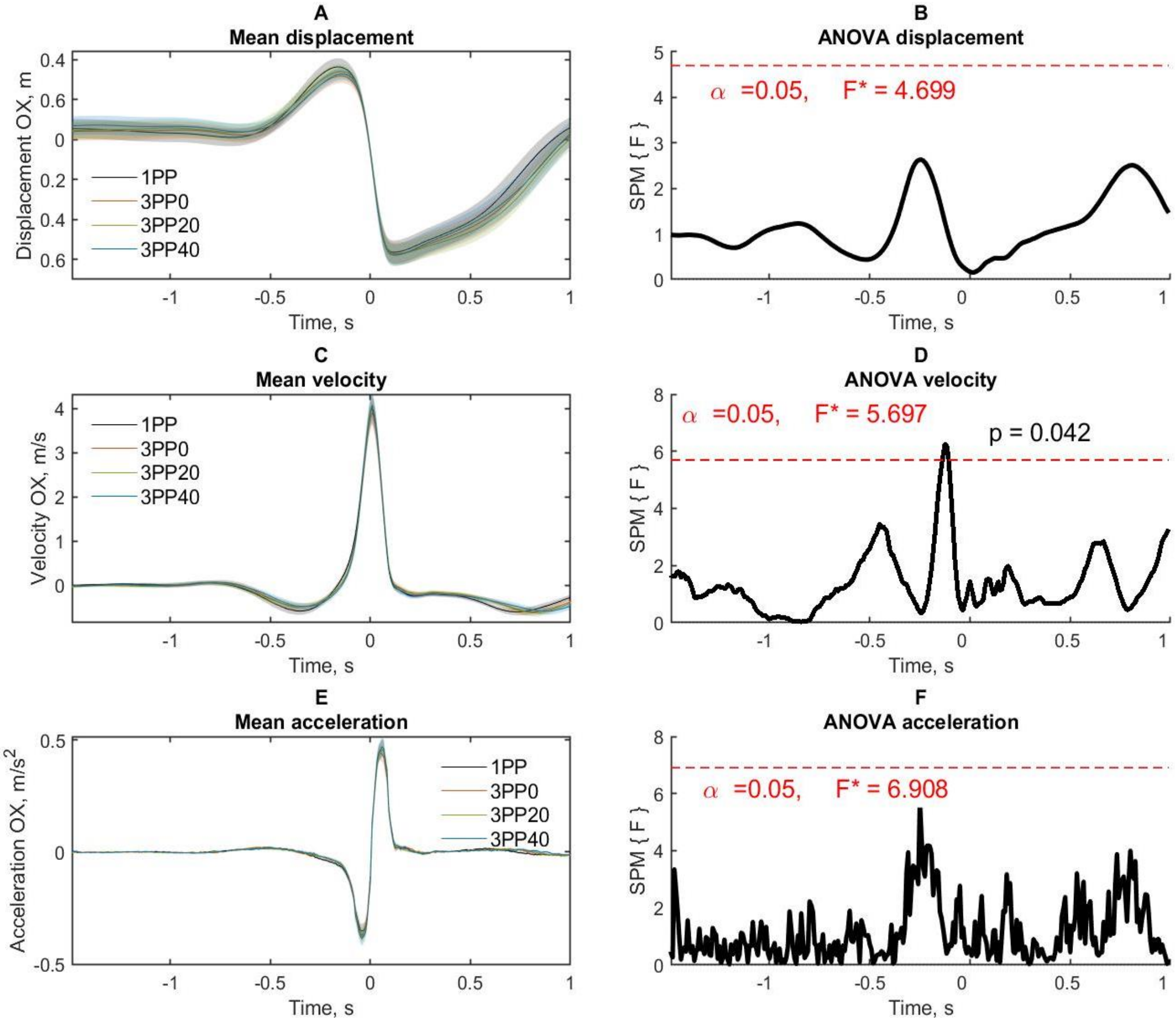

Figure S2. The right hand kinematic data along the OX axis. **A, C, E.** Displacement, velocity, acceleration of the right hand in four Conditions of throws. Semitransparent areas around lines are standard error of mean. **B, D, F.** Effect of Condition on ANOVA along the OX axis. Red dashed lines denotes the statistically significant level (with FWER correction). 1PP – first person condition, 3PP0 – third-person perspectives with 0°, 3PP20 – third-person perspectives with 20°, 3PP40 – third-person perspectives with 40°. Time 0 represents the moment of peak velocity of the right hand moving along the OY axis (along the direction of the throw).

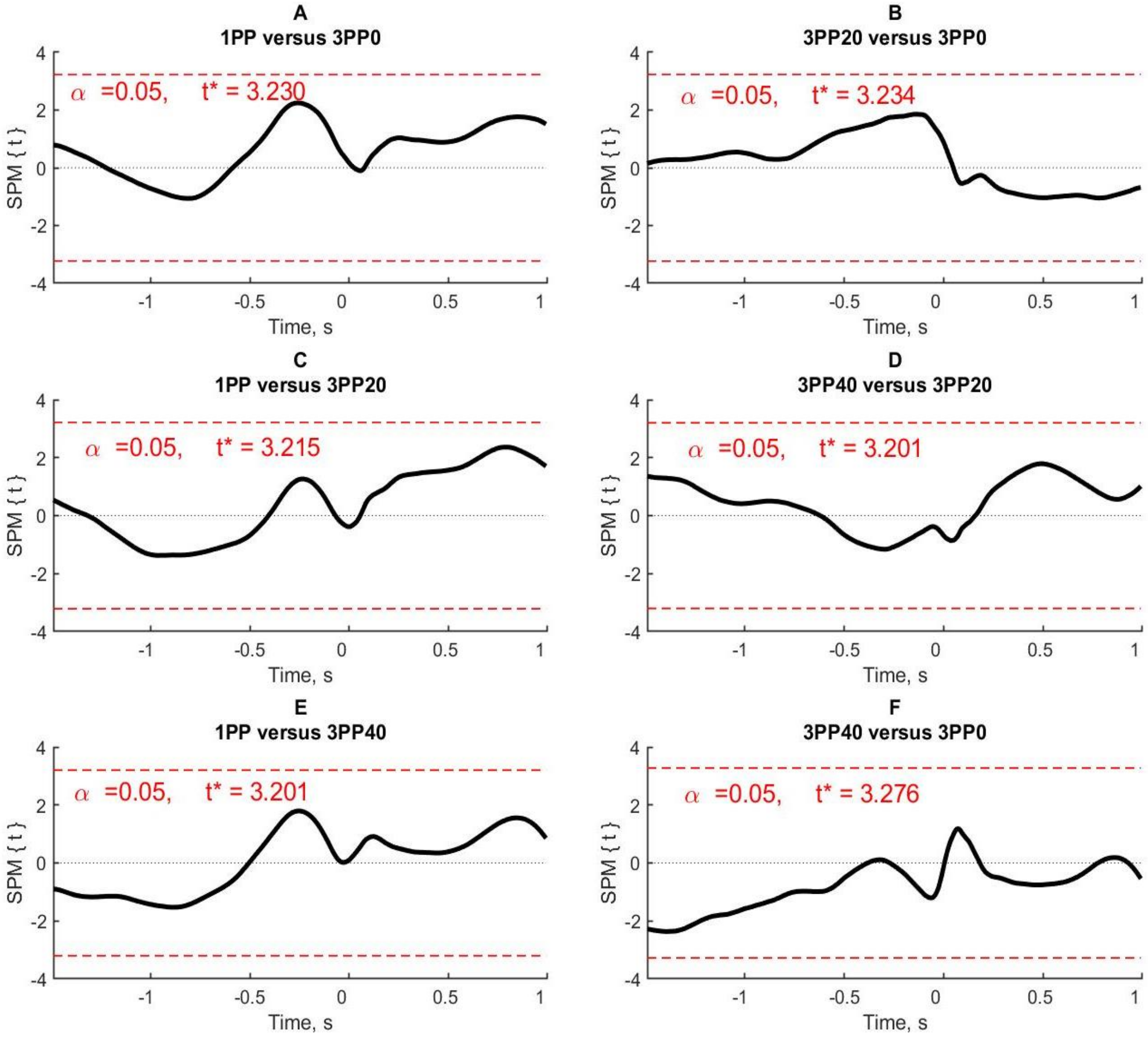

Figure S3. Pairwise comparisons mean displacement between conditions along the OX axis. Red dashed lines denotes the statistically significant level (with FWER correction). Time 0 represents the moment of peak velocity of the right hand moving along the OY axis (along the direction of the throw).

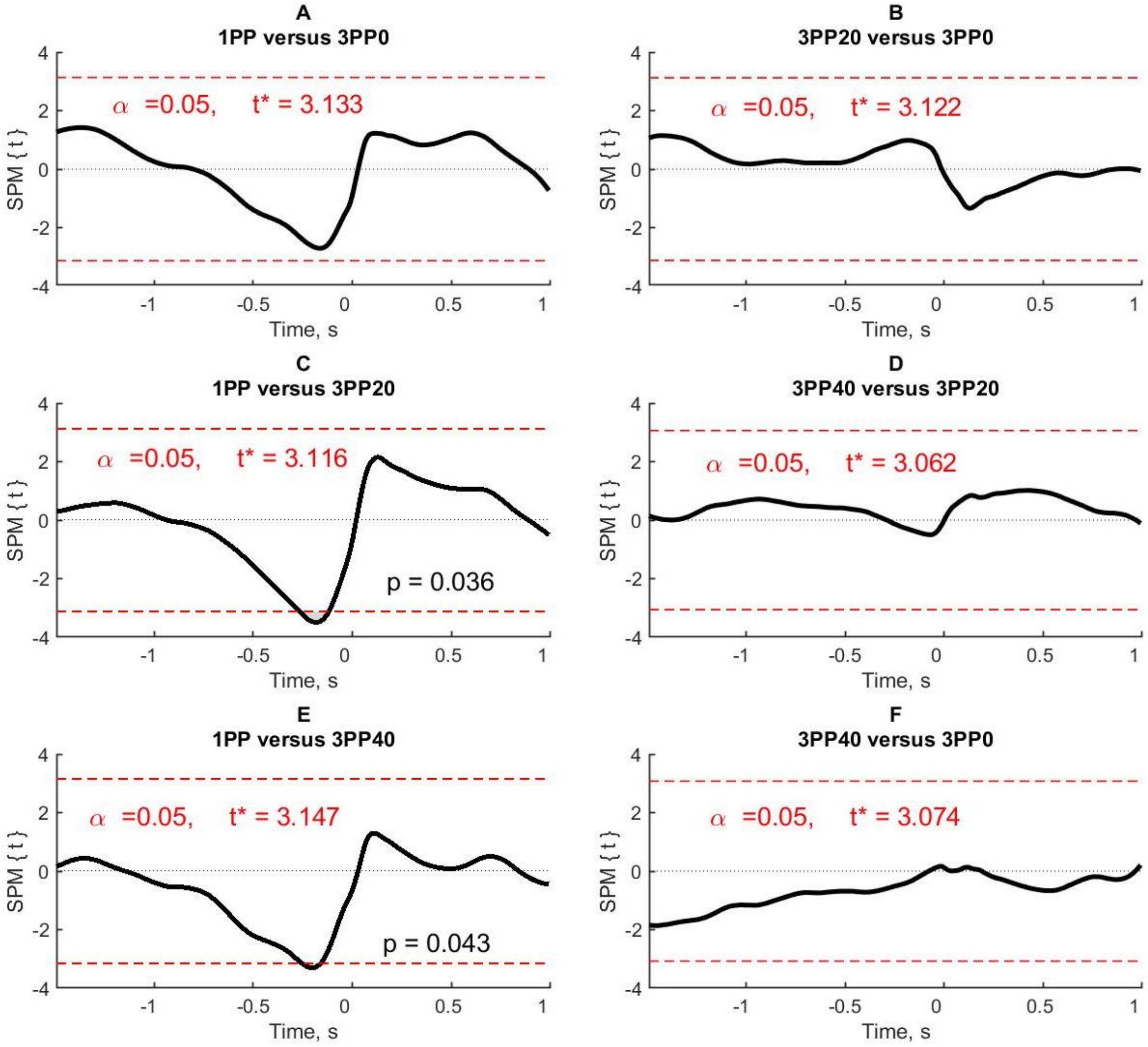

Figure S4. Pairwise comparisons mean displacement between conditions along the OY axis. Red dashed lines denotes the statistically significant level (with FWER correction). Time 0 represents the moment of peak velocity of the right hand moving along the OY axis (along the direction of the throw).

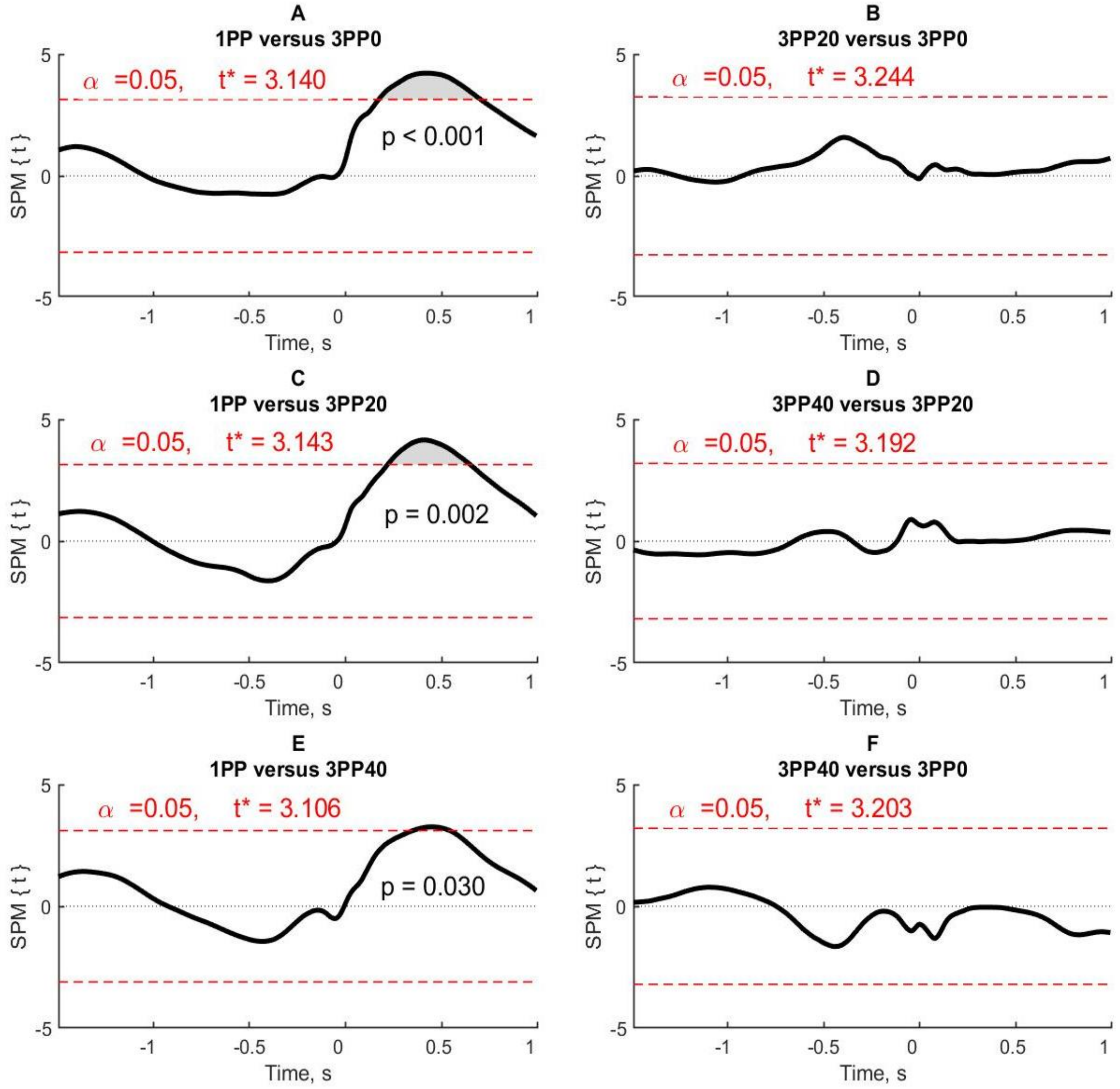

Figure S5. Pairwise comparisons mean displacement between conditions along the OZ axis. Red dashed lines denotes the statistically significant level (with FWER correction). Time 0 represents the moment of peak velocity of the right hand moving along the OY axis (along the direction of the throw).

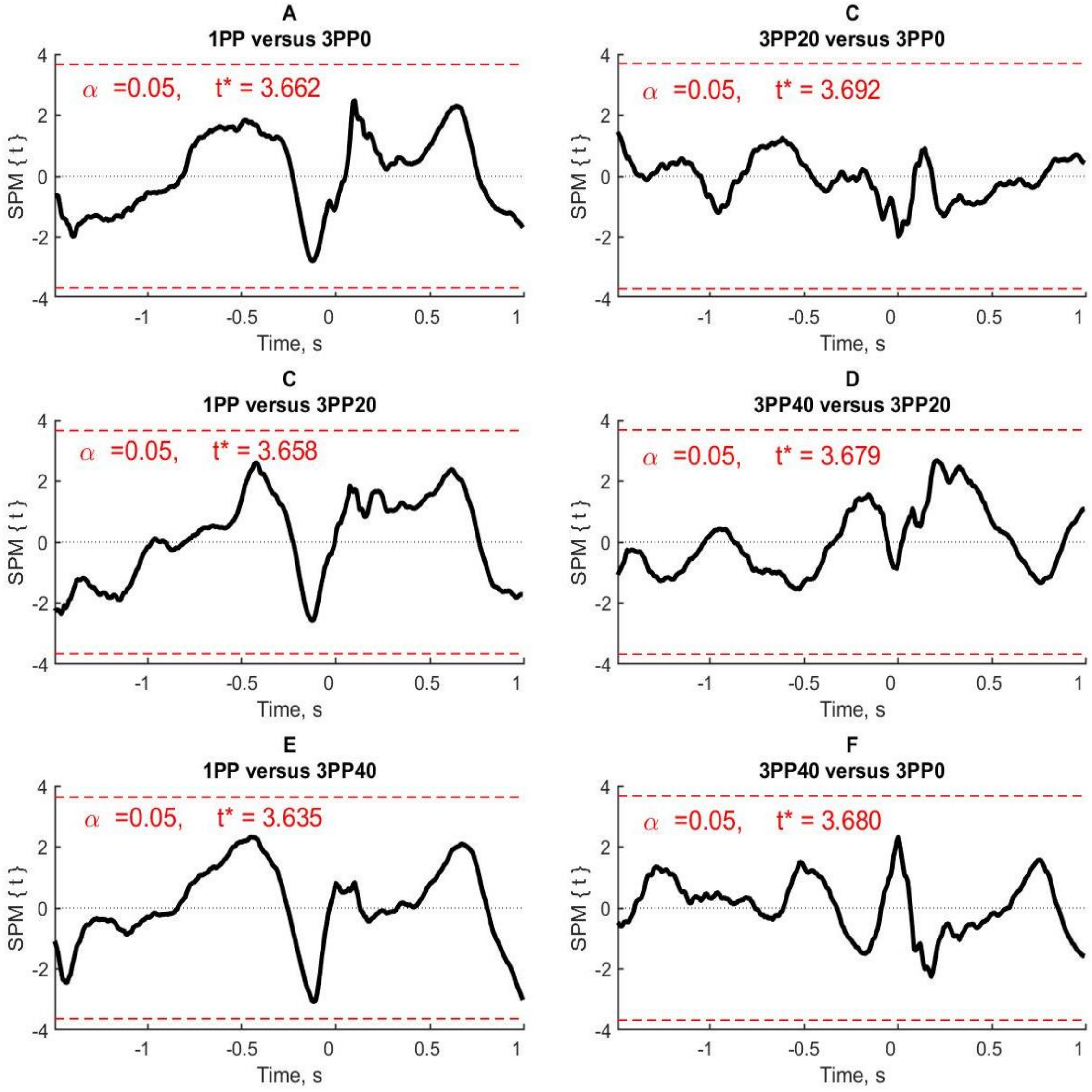

Figure S6. Pairwise comparisons mean velocity between conditions along the OX axis. Red dashed lines denotes the statistically significant level (with FWER correction). Time 0 represents the moment of peak velocity of the right hand moving along the OY axis (along the direction of the throw).

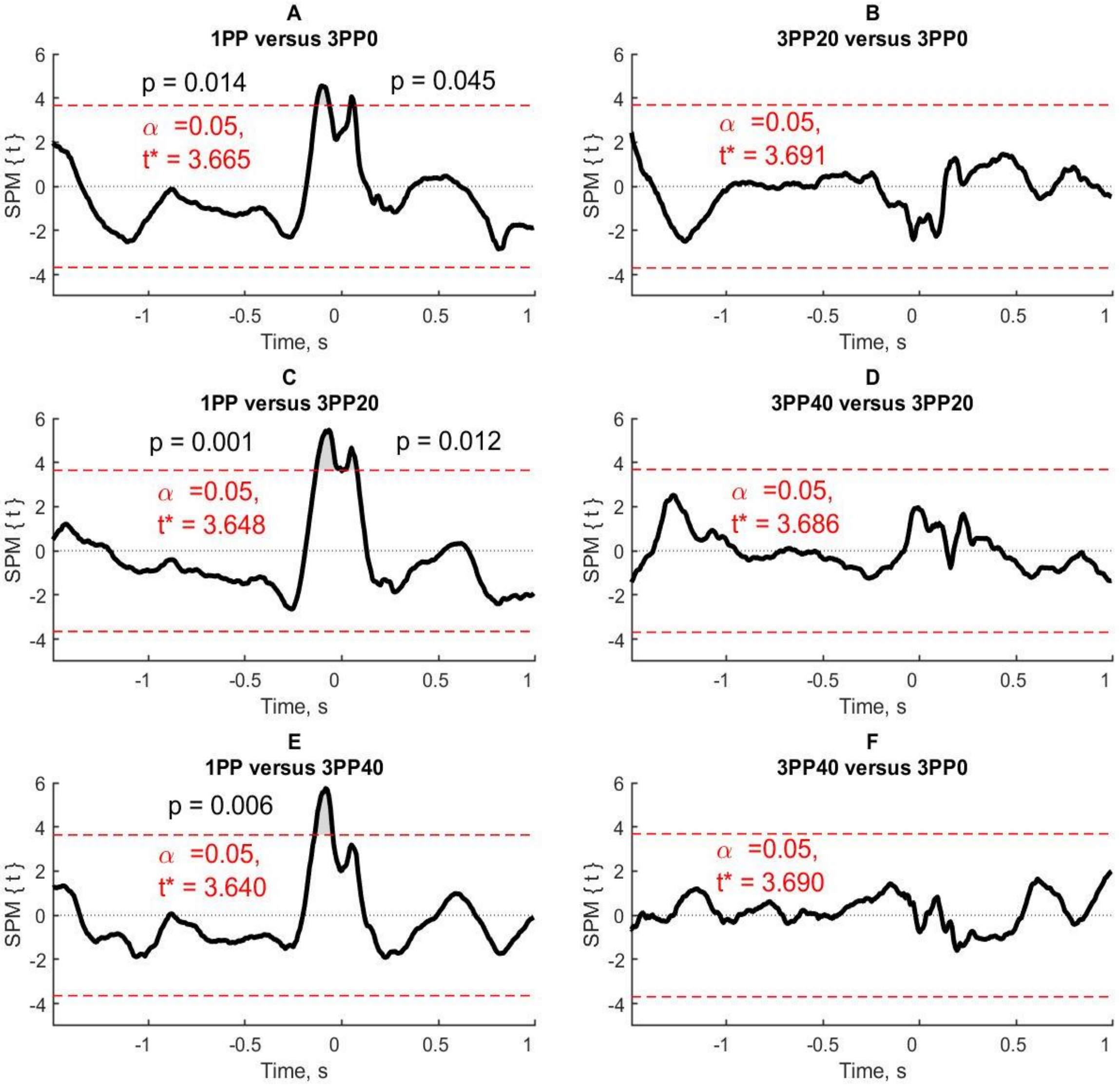

Figure S7. Pairwise comparisons mean velocity between conditions along the OY axis. Red dashed lines denotes the statistically significant level (with FWER correction). Time 0 represents the moment of peak velocity of the right hand moving along the OY axis (along the direction of the throw).

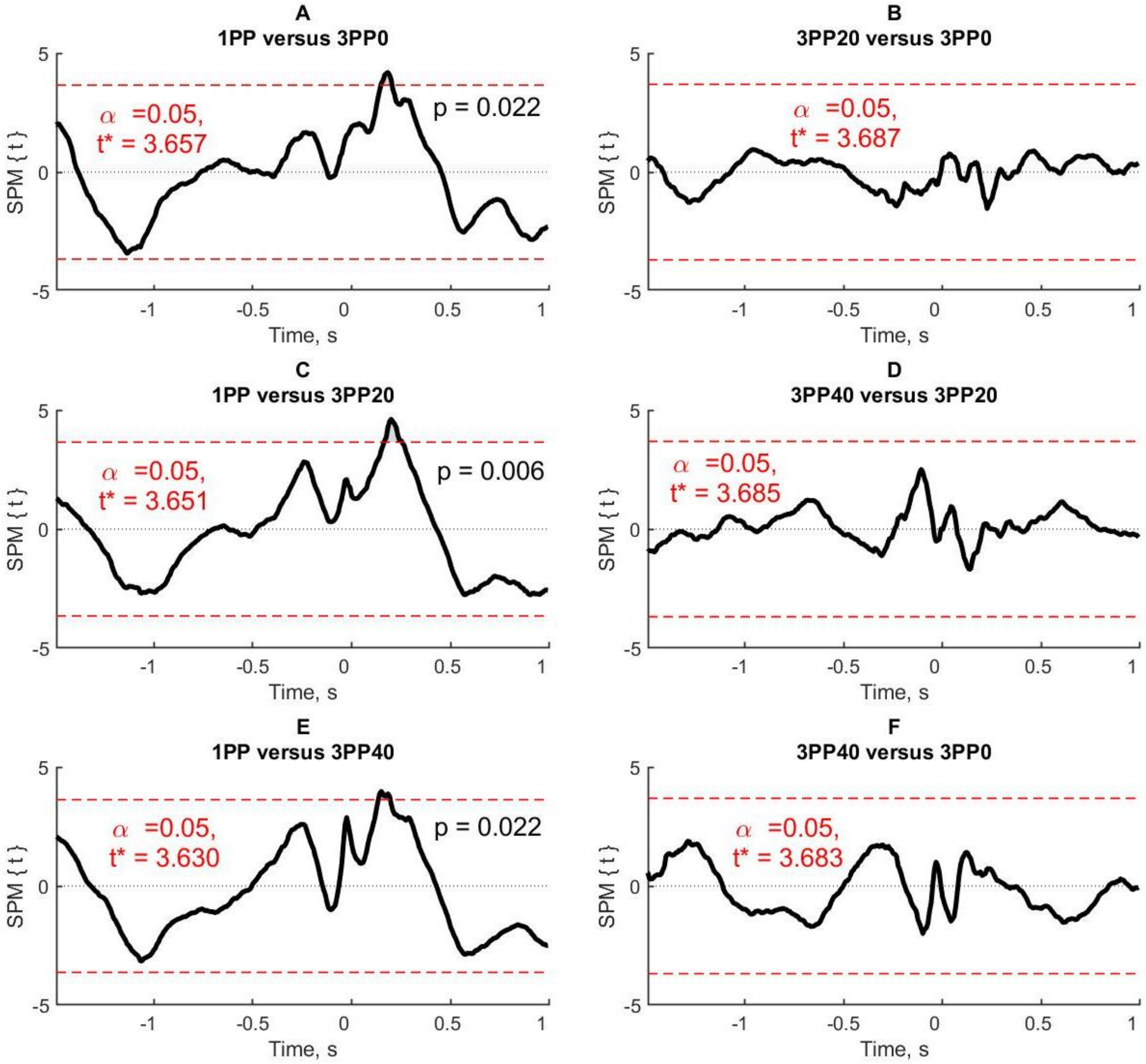

Figure S8. Pairwise comparisons mean velocity between conditions along the OZ axis. Red dashed lines denotes the statistically significant level (with FWER correction). Time 0 represents the moment of peak velocity of the right hand moving along the OY axis (along the direction of the throw).

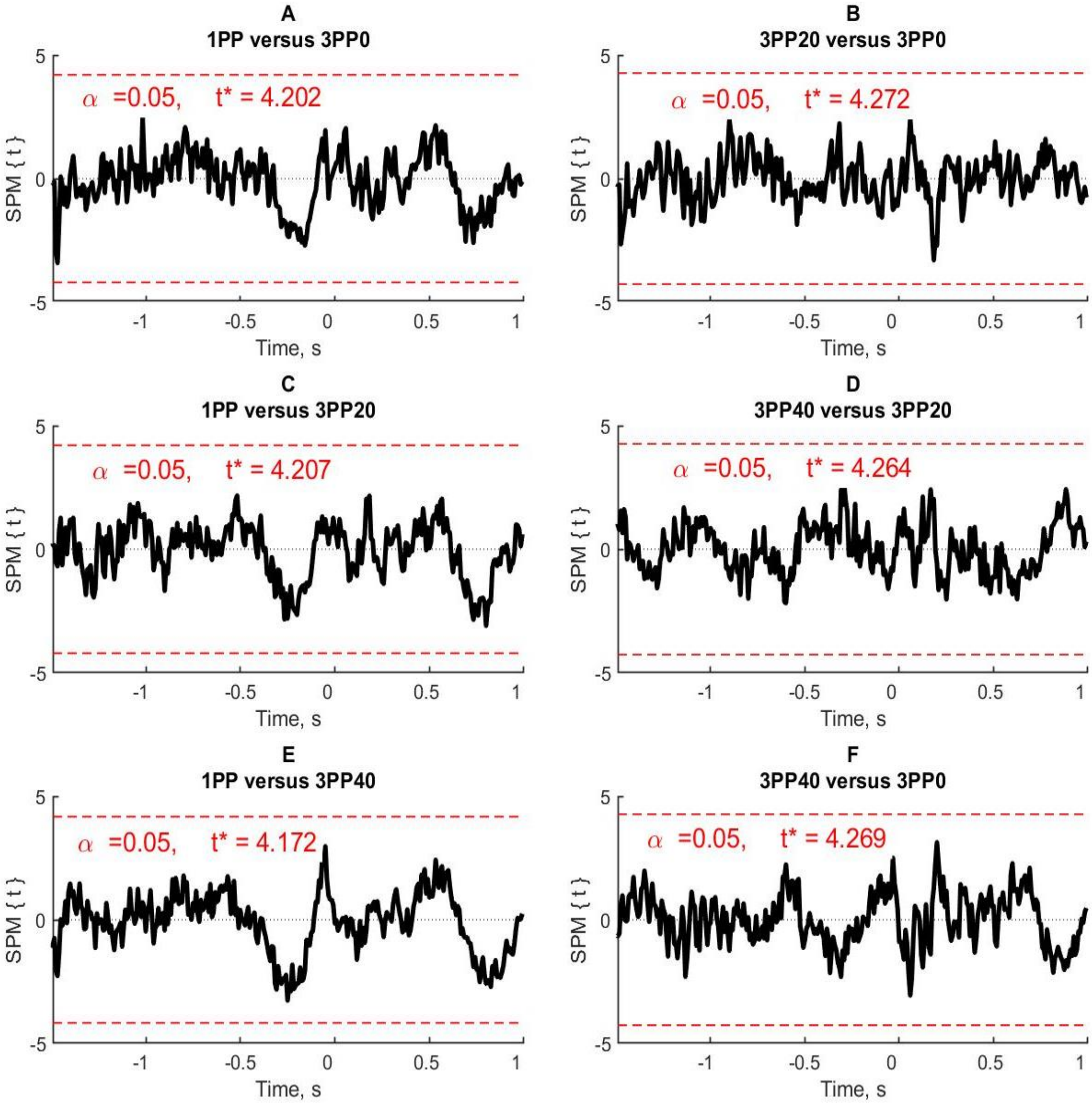

Figure S9. Pairwise comparisons mean acceleration between conditions along the OX axis. Red dashed lines denotes the statistically significant level (with FWER correction). Time 0 represents the moment of peak velocity of the right hand moving along the OY axis (along the direction of the throw).

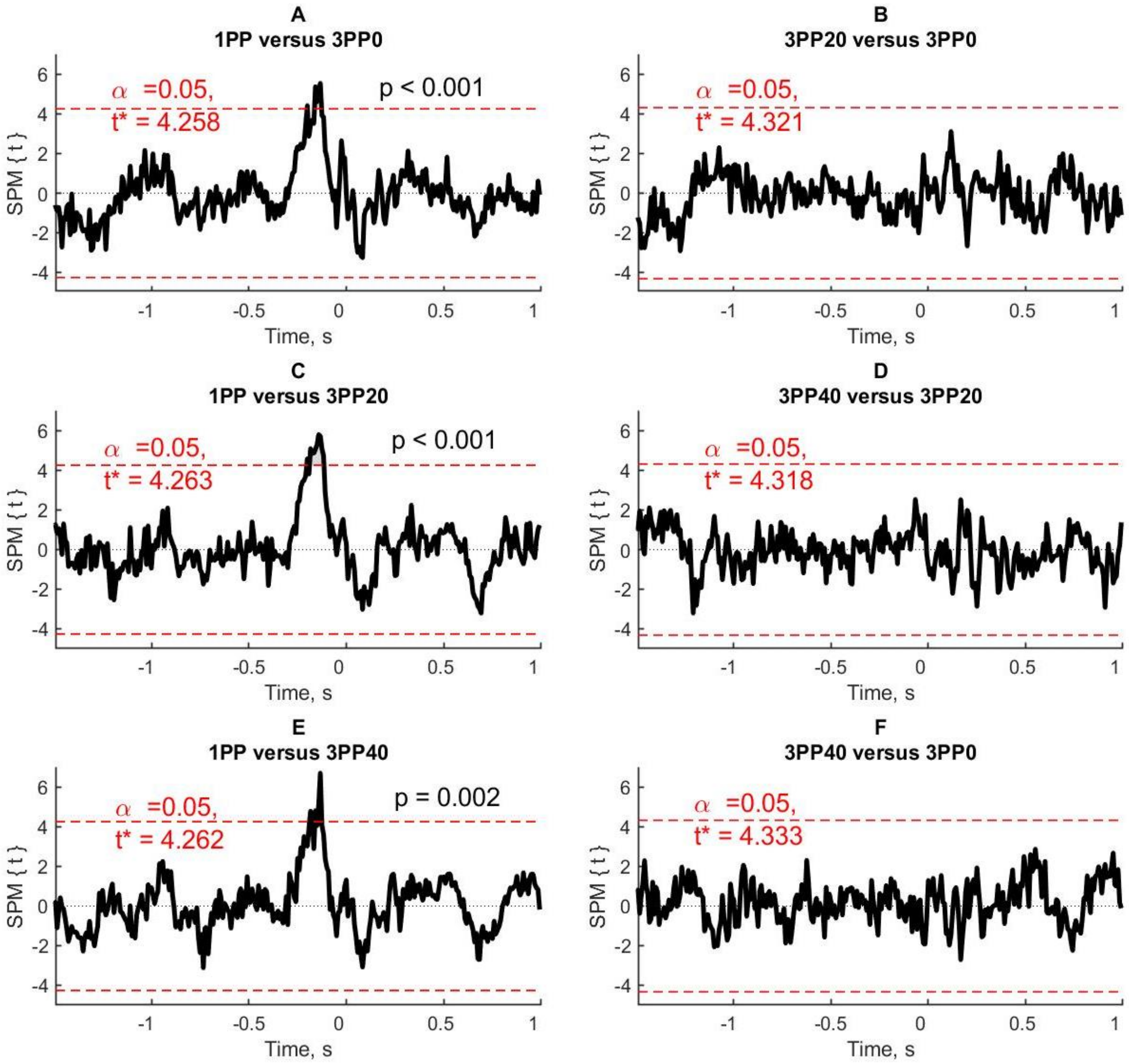

Figure S10. Pairwise comparisons mean acceleration between conditions along the OY axis. Red dashed lines denotes the statistically significant level (with FWER correction). Time 0 represents the moment of peak velocity of the right hand moving along the OY axis (along the direction of the throw).

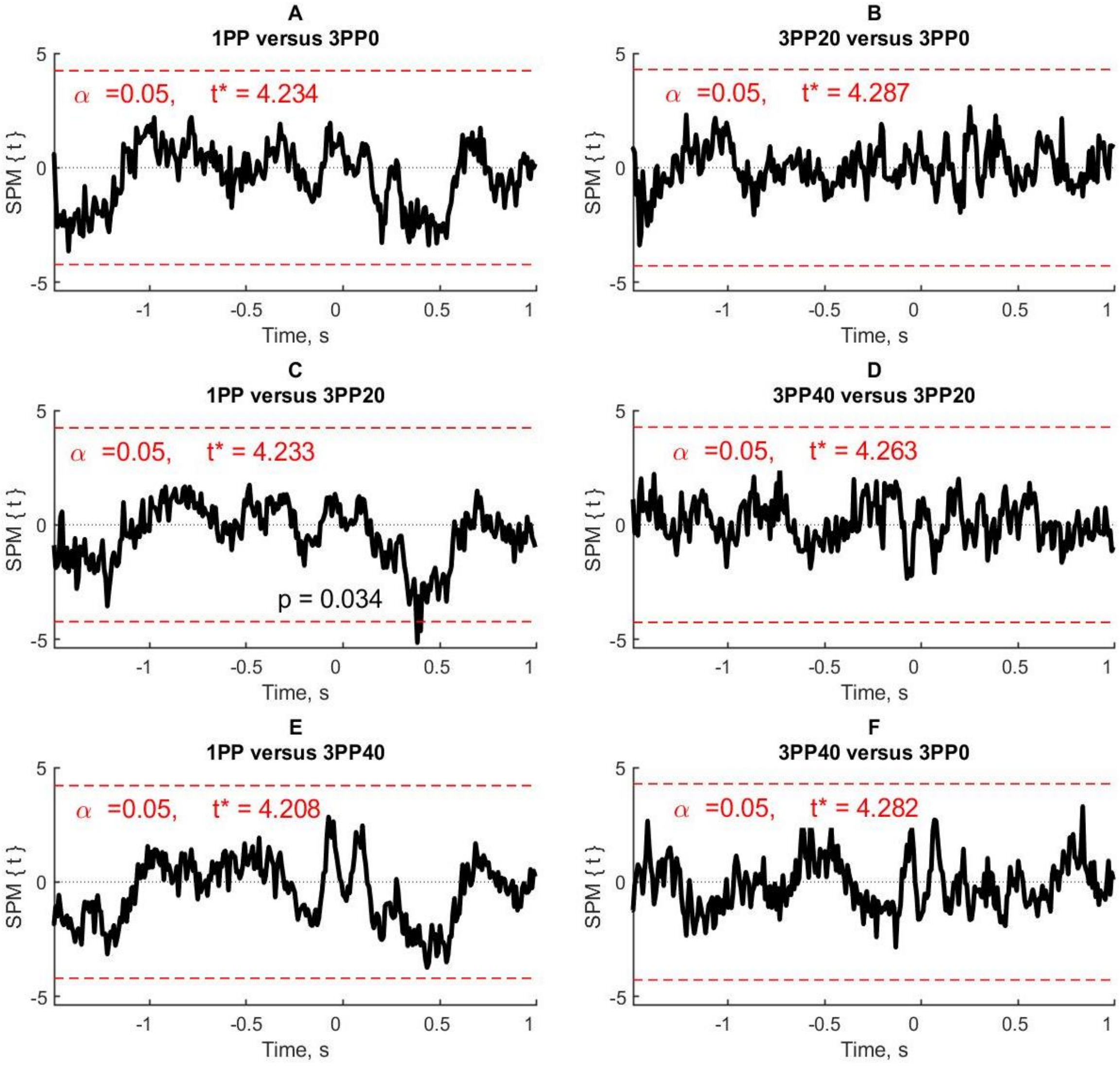

Figure S11. Pairwise comparisons mean acceleration between conditions along the OZ axis. Red dashed lines denotes the statistically significant level (with FWER correction). Time 0 represents the moment of peak velocity of the right hand moving along the OY axis (along the direction of the throw).

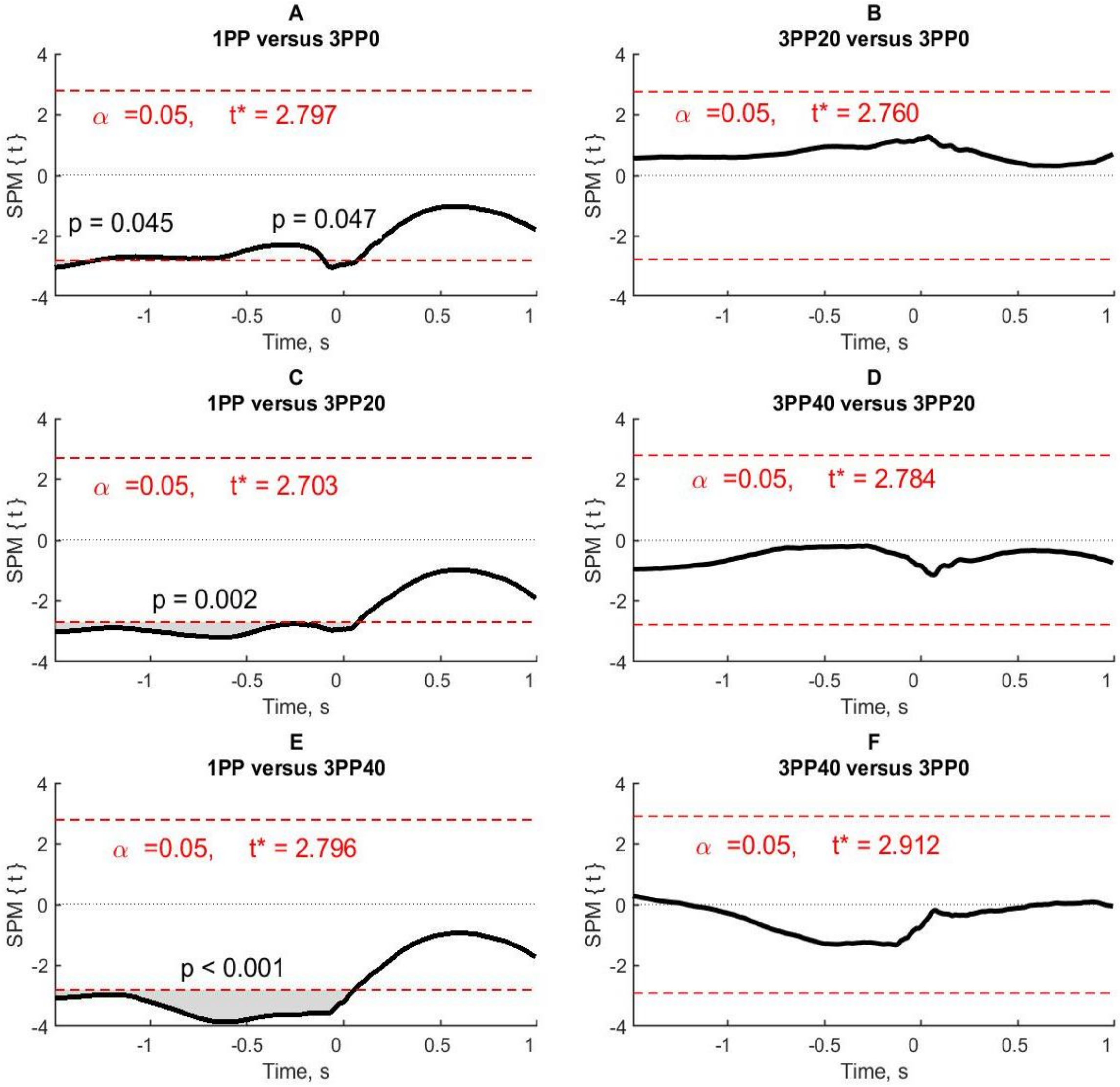

Figure S12. Pairwise comparisons mean angle torso rotation between conditions around the OZ axis. Red dashed lines denotes the statistically significant level (with FWER correction). Time 0 represents the moment of peak velocity of the right hand moving along the OY axis (along the direction of the throw).

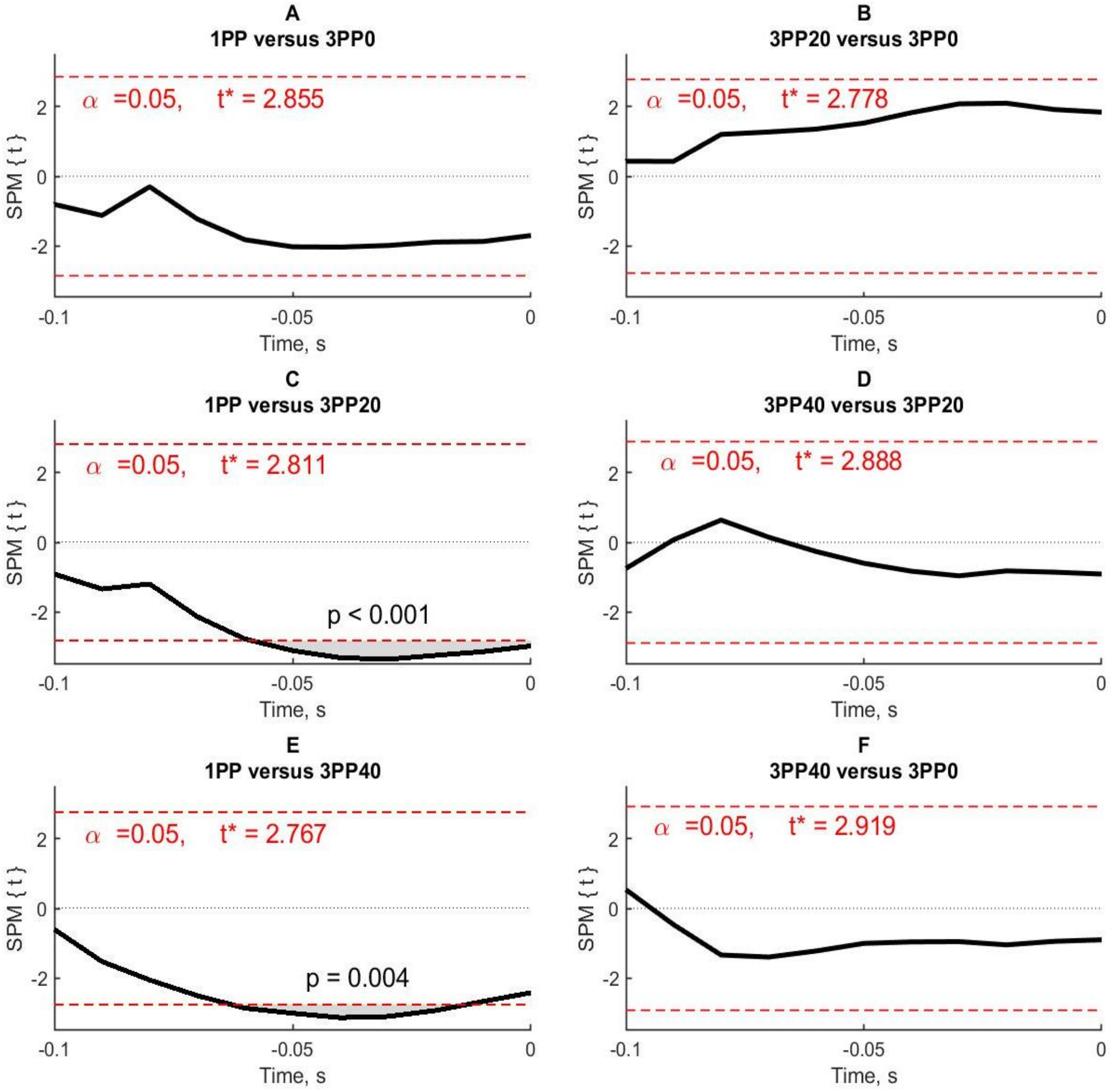

Figure S13. Pairwise comparisons mean right hand movement angle between conditions on the XY plane. Red dashed lines denotes the statistically significant level (with FWER correction). Time 0 represents the moment of peak velocity of the right hand moving along the OY axis (along the direction of the throw).
